# *EZH2*-triggered methylation of *SMAD3* promotes its activation and tumor metastasis

**DOI:** 10.1172/JCI152394

**Published:** 2022-03-01

**Authors:** Changsheng Huang, Fuqing Hu, Da Song, Xuling Sun, Anyi Liu, Qi Wu, Xiaowei She, Yaqi Chen, Lisheng Chen, Fayong Hu, Feng Xu, Xuelai Luo, Yongdong Feng, Xiangping Yang, Junbo Hu, Guihua Wang

**Affiliations:** 1 GI Cancer Research Institute, Tongji Hospital, Huazhong University of Science and Technology, Wuhan, China.; 2 Department of General Surgery, First Affiliated Hospital, School of Medicine, Shihezi University, Shihezi, Xinjiang, China.; 3 Department of Immunology, Tongji Medical College, Huazhong University of Science and Technology, Wuhan, China.

**Keywords:** Oncology, Cancer, Molecular biology

## Abstract

*SMAD3* plays a central role in cancer metastasis, and its hyperactivation is linked to poor cancer outcomes. Thus, it is critical to understand the upstream signaling pathways that govern *SMAD3* activation. Here, we report that *SMAD3* underwent methylation at K53 and K333 (K53/K333) by *EZH2*, a process crucial for cell membrane recruitment, phosphorylation, and activation of *SMAD3* upon *TGFB1* stimulation. Mechanistically, *EZH2*-triggered *SMAD3* methylation facilitated *SMAD3* interaction with its cellular membrane localization molecule (*SARA*), which in turn sustained *SMAD3* phosphorylation by the *TGFB* receptor. Pathologically, increased expression of *EZH2* expression resulted in the accumulation of *SMAD3* methylation to facilitate *SMAD3* activation. *EZH2*-mediated *SMAD3* K53/K333 methylation was upregulated and correlated with *SMAD3* hyperactivation in breast cancer, promoted tumor metastasis, and was predictive of poor survival outcomes. We used 2 TAT peptides to abrogate *SMAD3* methylation and therapeutically inhibit cancer metastasis. Collectively, these findings reveal the complicated layers involved in the regulation of *SMAD3* activation coordinated by *EZH2*-mediated *SMAD3* K53/K333 methylation to drive cancer metastasis.

## Introduction

Tumor metastasis is highly responsible for tumor-related death (1–4). During metastasis, epithelial cells gradually discard their differentiated characteristics, including losing their cellular polarity and cell-cell adhesion capacity, thereby gaining mesenchymal characteristics such as invasion, migration, and motility, which is also referred to as epithelial-mesenchymal transition (EMT) (5, 6). *TGFB* plays a key role in development, inflammation, homeostasis, and multiple diseases, including tumor metastasis and EMT (4, 7–9). In premalignant cells, *TGFB* acts as a potent tumor suppressor by inhibiting cell proliferation, promoting apoptosis, and maintaining genome stability (10, 11). However, *TGFB* signaling orchestrates the EMT, and cancer cells use *TGFB* to create an immune-suppressive tumor microenvironment (TME), which suppresses the antitumor immune reactions that foster tumor progression and metastasis (1, 2, 5, 9, 12–15). The cellular effect induced by *TGFB* occurs via specific type I and II serine/threonine kinase receptors, followed by *SMAD2* and *SMAD3* (R-*SMAD*s) C-terminal phosphorylation and then formation of a complex with *SMAD4* (16). *SMAD3*, one of the receptor-regulated R-*SMAD*s, is directly phosphorylated at the C-terminal SSXS motif by the *TGFB* type I receptor (*TBRI*) and plays an essential role in the canonical *TGFB* signaling pathway (17). Following the binding of R-*SMADs* to common *SMAD*, the complex is translocated into the nucleus, where it binds to transcriptional coactivators or corepressors and regulates transcription (15). Interestingly, Recent studies show that the biological function of *SMAD3* opposes that of *SMAD2* in tumor metastasis, and silencing *SMAD2* promotes tumor metastasis, whereas *SMAD3* KO inhibits tumor metastasis (18, 19). Given these confusing phenomena, the mechanism underlying the regulation of *TGFB* signaling merits in-depth investigation.

Posttranslational modifications (PTMs) of nonhistone proteins, such as phosphorylation, acetylation, methylation, and ubiquitination, are involved in protein stability, catalytic activity, and protein-protein interaction (20, 21). Dysregulation of these modifications induced severe diseases including cancer (22). Recently, lysine methylation of nonhistone proteins has been identified as a prevalent PTM (23–26). *SMAD3* is subject to extensive PTMs, including phosphorylation, ubiquitination, and acetylation, which are important for *SMAD3* activation, translocation, and stability (27–33). However, whether lysine methylation of the key protein *SMAD3* in the *TGFB1* signaling pathway plays a crucial role in *TGFB*/*SMAD*s signaling activation has, to our knowledge, yet to be investigated.

Here, we report a PTM for *SMAD3*, methylation. We show that K53 and K333 (K53/K333)methylation of *SMAD3* was crucial for *SMAD3* cell membrane recruitment, phosphorylation, and biological function, stimulated by *TGFB1*. Deletion of *SMAD3* K53/K333 methylation can dramatically inhibit tumor metastasis. Therefore, this study reveals that *EZH2*-mediated *SMAD3* K53/K333 methylation was necessary for *SMAD3* transcriptional activity and that targeting *SMAD3* K53/K333 methylation might offer a potential therapeutic strategy for the treatment of *TGFB*/*SMAD* tumors.

## Results

### Methylation of SMAD3 is essential for TGFB1-mediated SMAD3 activity.

**Figure 1:**
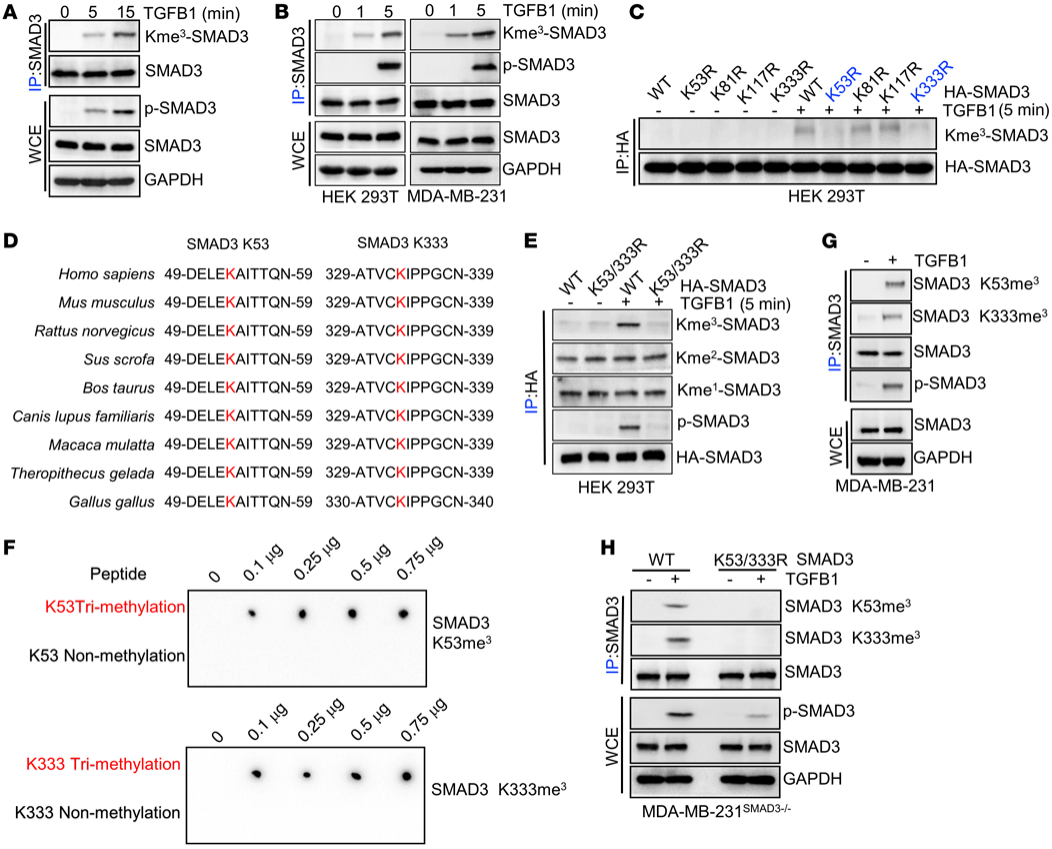
*SMAD3* K53/K333 methylation is critical for *SMAD3* activation. (**A** and **B**) HEK293T cells and MDA-MB-231 cells were serum starved and treated with *TGFB1* (5 ng/mL) for the indicated durations, and whole-cell extracts (WCEs) were collected for IP with anti-SMAD3 antibody, followed by immunoblot (IB) analysis. (**C**) HEK293T cells were transfected with WT *HA*-*SMAD3* or mutant plasmids as indicated and treated with *TGFB1* (5 ng/mL). WCEs were then collected for IP with anti-HA antibody, followed by IB analysis. (**D**) *SMAD3* K53/K333 site aa in different species. (**E**) HEK293T cells were transfected with WT *HA*-*SMAD3* or K53/333R-mutant plasmids and then treated with *TGFB1* (5 ng/mL). WCEs were collected for IP with anti-HA antibody, followed by IB analysis. (**F**) ddH_2_O (10 μL) containing different peptides (0.1–0.75 μg) was added onto the PVDF membranes, followed by IB analysis using a K53-specific trimethylation antibody (anti–SMAD3 K53me3) and a K333-specific trimethylation antibody (anti–SMAD3 K333me3). (**G**) MDA-MB-231 cells were serum starved and treated with *TGFB1* (5 ng/mL), and WCEs were collected for IP with anti-SMAD3 antibody, followed by IB analysis with *SMAD3* K53/K333 trimethylation–specific antibodies. (**H**) MDA-MB-231^SMAD3–/–^ cells were stably transfected with WT *SMAD3* or *SMAD3* K53/333R plasmids and treated with *TGFB1* (5 ng/mL). WCEs were collected for IP with anti-SMAD3 antibody, followed by IB analysis. All immunoblotting was performed 3 times, independently, with similar results.

To identify potentially novel *SMAD3* PTMs, we first performed a co-IP assay to isolate *SMAD3* protein from HEK293T cells and then conducted mass spectrometric analysis. Interestingly, we found that methylation was one of the potential *SMAD3* PTMs (Supplemental Table 1; supplemental material available online with this article; https://doi.org/10.1172/JCI152394DS1). Of note, our mass spectrometric analysis led us to other studies that also reported *SMAD3* PTMs, such as phosphorylation (Supplemental Table 1). Protein lysine methylation has been identified as a prevalent PTM and is an important regulator of cellular signal transduction. To prove the mass spectrometry analysis and further investigate whether the lysine of *SMAD3* can be methylated, we carried out a co-IP assay to isolate the *SMAD3* protein from HEK293T cells and then used the pan–lysine trimethylation (pan–K-me3) antibody for detection. Notably, we observed that the lysine of *SMAD3* could be trimethylated (Supplemental Figure 1A). Moreover, the trimethylation levels, but not the di- or monomethylation levels, of *SMAD3* were increased by *TGFB1* stimulation (Figure 1A and Supplemental Figure 1, B and C). *SMAD3* phosphorylation at S423/S425 (*SMAD3*^S423/S425^ phosphorylation) was necessary for its transcriptional activity. Next, to determine whether there is a relationship between *SMAD3* lysine trimethylation and *SMAD3*^S423/S425^ phosphorylation, we shortened the duration of *TGFB1* stimulation of HEK293T cells. Importantly, upon *TGFB1* stimulation, we found that lysine trimethylation of *SMAD3* occurred before *SMAD3*^S423/S425^ phosphorylation (Supplemental Figure 1D and Figure 1B). Furthermore, the global histone methylation inhibitor 3-deazaneplanocin A (DZneP) (34) inhibited *SMAD3* lysine trimethylation and *SMAD3*^S423/S425^ phosphorylation (Supplemental Figure 1E). On the basis of our previous mass spectrometric results, we hypothesized that lysines 53, 81, 117, and 333 of *SMAD3* might be potential methylation sites (Supplemental Figure 1F).

To further investigate the methylation sites of *SMAD3* upon *TGFB1* stimulation, we constructed methylation-deficient variants of *SMAD3* (K53R, K81R, K117R, and K333R), which were expressed in HEK293T cells with or without *TGFB1* stimulation. The data showed that only the K53R and K333R variants partly abrogated the trimethylation upregulation of *SMAD3* under *TGFB1* stimulation (Figure 1C). Of note, the K53 and K333 sites were highly conserved among various species (Figure 1D). Moreover, the special methylation-deficient variant of *SMAD3*, K53/333R, completely abrogated the trimethylation upregulation of *SMAD3* and dramatically inhibited *SMAD3*^S423/S425^ phosphorylation under *TGFB1* stimulation (Figure 1E). Next, we generated a K53-specific trimethylation antibody (anti–SMAD3 K53me^3^) and a K333-specific trimethylation antibody (anti–SMAD3 K333me^3^), which specifically recognized *SMAD3* K53/K333 trimethylation using a dot-blot assay (Figure 1F). In addition, IHC analysis of lung metastasis led by MDA-MB-231 *SMAD3* WT and KO cell lines further confirmed that anti–phosphorylated SMAD3^S423/S425^ (anti–p-SMAD^S423/S425^) , anti–*SMAD3* K53me^3^, and anti–SMAD3 K333me^3^ antibodies specifically recognized *SMAD3*^S423/S425^ phosphorylation and K53/K333 trimethylation (Supplemental Figure 2I). Using the anti–SMAD3 K53me^3^ and anti–*SMAD3* K333me^3^ antibodies, we further confirmed that *SMAD3* K53/K333 trimethylation was induced by *TGFB1* treatment (Figure 1G). Next, we generated a *SMAD3*-KO MDA-MB-231 cell line using the CRISPR/Cas9 system (Supplemental Figure 1H) and subsequently induced ectopically stable expression of a methylation-deficient variant of *SMAD3*, K53/333R, in *SMAD3*-KO MDA-MB-231 cells (Supplemental Figure 1I). Compared with *SMAD3* WT, we found that *SMAD3* K53/333R dramatically reduced *SMAD3*^S423/S425^ phosphorylation under *TGFB1* stimulation (Figure 1H). Therefore, *SMAD3* K53/K333 trimethylation was induced by *TGFB1* stimulation and necessary for *SMAD3*^S423/S425^ phosphorylation.

### Absence of SMAD3 methylation inhibits its oncogenic functions in vivo and in vitro.

**Figure 2:**
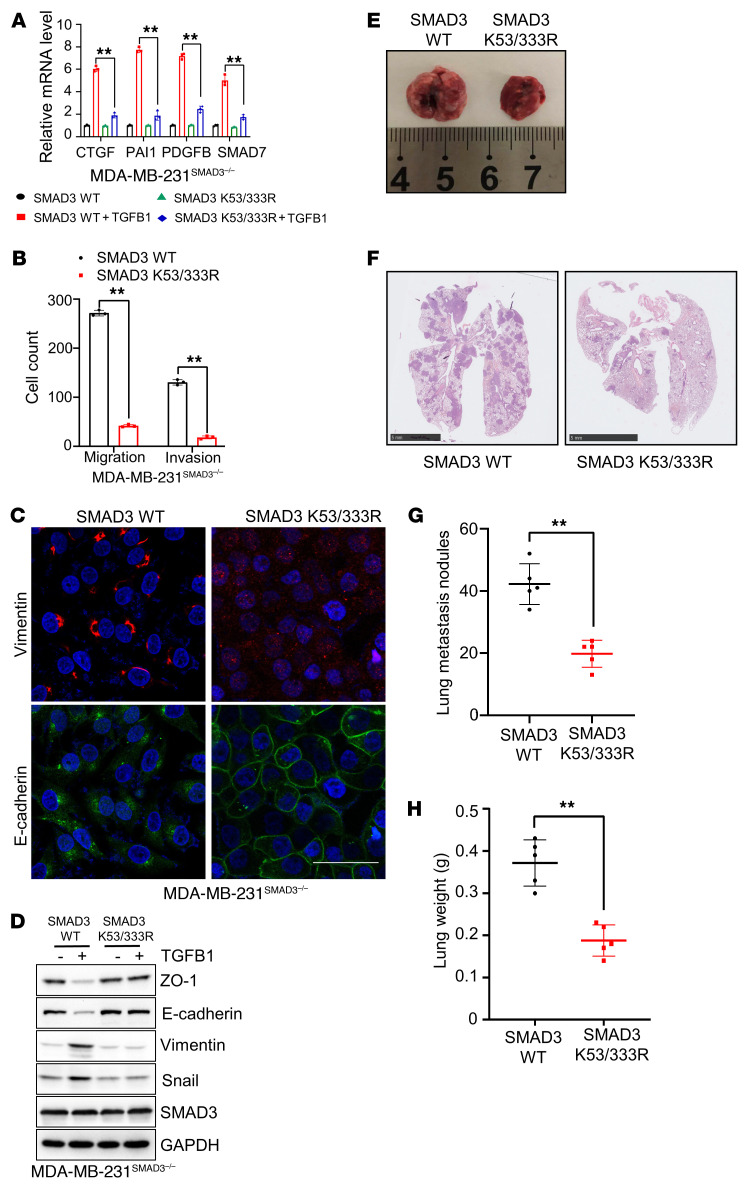
Deletion of *SMAD3* K53/K333 methylation inhibits the *SMAD3* oncogene in vitro and in vivo. (**A**) MDA-MB-231^SMAD3–/–^ cells were stably transfected with WT *SMAD3* or *SMAD3* K53/333R plasmids and treated with *TGFB1* (5 ng/mL). Quantitative RT-PCR analysis of *TGFB*/*SMAD3* signaling pathway downstream genes, including *CTGF*, *PAI1*, *PDGFB*, and *SMAD7*, in the indicated cells with or without *TGFB1* (5 ng/mL) treatment. (**B**) Quantitative analysis of Transwell assay in the indicated cells. (**C**) IF and (**D**) IB analysis of EMT markers in the indicated cells. Scale bar: 50 um. (**E** and **F**) Representative lung image (**E**) and H&E-stained lung sections (**F**). Scale bars: 5 mm. (**G** and **H**) Scatter plots showing lung metastatic nodules (**G**) and lung weights (**H**). All immunoblots were performed 3 times, independently, with similar results. Data indicate the mean ± SD. **P < 0.05, by 2-tailed Student’s *t* test (**A**, **B**, **G**, and **H**).

*TGFB* is a potent inducer of EMT, whereby epithelial progenitor cells lose polarity, downregulate cell-cell adhesions, and migrate and invade to generate or regenerate tissues (14, 35). Beyond the contribution of *TGFB*-induced EMT to tumor invasion and metastatic dissemination, the *TGFB* pathway induces gene responses that support the ability of cancer cells to infiltrate and colonize specific organs (19). To reveal the potential biological functions of *SMAD3* K53/K333 methylation and their role in *TGFB* pathway activation, we first measured the mRNA levels of *CTGF*, *PAI1*, *PDGFB*, and *SMAD7*, which are transcriptionally regulated by the *SMAD2*-*SMAD3* complex and are predicted to activate the canonical *TGFB* pathway activation in cells with or without *TGFB1* treatment (11, 36). The results showed that *SMAD3* K53/333R cells had significantly lower mRNA levels of *CTGF*, *PAI1*, *PDGFB*, and *SMAD7* compared with levels in *SMAD3* WT cells upon *TGFB1* treatment (Figure 2A and Supplemental Figure 2, A and B). We then showed that the migratory and invasive abilities of *SMAD3* K53/333R cells dramatically decreased compared with what we observed with *SMAD3* WT cells (Figure 2B and Supplemental Figure 2C). Furthermore, *SMAD3* K53/333R cells had higher protein expression of ZO-1 and E-cadherin and lower protein expression of vimentin and snail compared with *SMAD3* WT cells (Figure 2, C and D). Under the *TGFB1* stimulation condition, the protein expression levels of ZO-1, E-cadherin, vimentin, and snail in *SMAD3* K53/333R cells showed no visual change compared with expression levels in *SMAD3* WT cells (Figure 2D). These observations indicate that *TGFB1*-induced *SMAD3* K53/333 methylation is critical for *SMAD3* function and *TGFB* pathway activation. To determine whether methylation-deficient mutants of *SMAD3* abrogate tumor metastasis in vivo, we injected *SMAD3* WT and *SMAD3* K53/333R MDA-MB-231 cells into the tail veins of 4-week-old female BALB/c nude mice. The results showed that *SMAD3* K53/333R cells generated fewer metastatic nodules and lower tumor weights than did *SMAD3* WT cells (Figure 2, E–H). Furthermore, the biological function of *SMAD3* K53/333R cells phenocopied that of *SMAD3*-KO cells (Supplemental Figure 2, D–H). Taken together, these results revealed that the methylation of *SMAD3* on K53 and K333 was crucial for *TGFB*/*SMAD* signaling pathway activation and the oncogenic functions of *SMAD3*.

### EZH2 methylates SMAD3 and promotes its activation.

Based on the finding that K53/333 methylation of *SMAD3* was essential for *TGFB*/*SMAD* signaling pathway activation and oncogenic functions, we then sought to determine which methyltransferase(s) mediated *SMAD3* K53/333 methylation. Given our observation of K53 and K333 upregulation and *TGFB1*-induced *SMAD3* trimethylation, we hypothesized that the physical interaction between potential methyltransferase(s) and *SMAD3* should also be enhanced by *TGFB1* stimulation. We transfected *HA-SMAD3* or a plasmid vector control into HEK293T cells and then pulled down *HA-SMAD3* using an anti-HA antibody under *TGFB1* treatment and nontreatment conditions. The pulled-down proteins were then subjected to systematic mass spectrometric analysis to identify potential methyltransferase(s) that may have triggered *SMAD3* K53/333 methylation.

**Figure 3:**
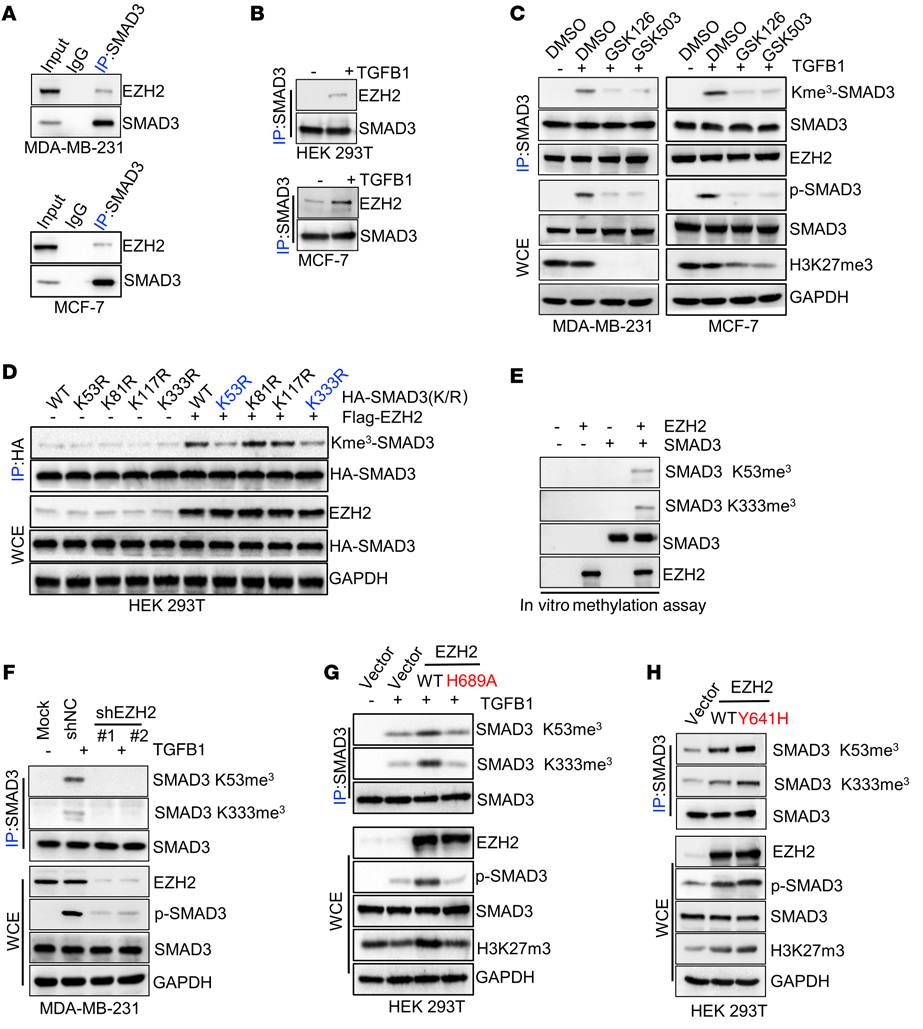
*SMAD3* methylation is triggered by *EZH2*. (**A**) WCEs of MDA-MB-231 and MCF-7 cells were collected and subjected to co-IP and IB assays. (**B**) HEK293T and MCF-7 cells were serum starved and treated with *TGFB1* (5 ng/mL), and WCEs were collected for IP with anti-SMAD3 antibody, followed by IB analysis. (**C**) MDA-MB-231 and MCF-7 cells were treated with *TGFB1* (5 ng/mL) and the *EZH2* inhibitors GSK126 or GSK503, and WCEs were collected for IP with anti-SMAD3 antibody, followed by IB analysis. (**D**) HEK293T cells were transfected with WT *HA-SMAD3* or mutant plasmids and a *Flag-EZH2* plasmid as indicated/WCEs were then collected for IP with anti-HA antibody, followed by IB analysis. (**E**) Immunoprecipitated *EZH2* from HEK293 cells was incubated with SAM along with *SMAD3* protein for in vitro methylation of *SMAD3*. The methylated proteins were separated by SDS-PAGE, and *SMAD3* methylation was analyzed by IB using anti–SMAD3 K53/K333 trimethylation–specific antibodies. (**F**) MDA-MB-231 cells silenced with control (shNC) or *EZH2* shRNA (nos. 1 and 2) were treated with *TGFB1* (5 ng/mL), and WCEs were collected for IP with anti-SMAD3 antibody, followed by IB analysis. (**G**) HEK293T cells were transfected with vector, *EZH2*^WT^, or *EZH2*^H689A^ and then treated with *TGFB1* (5 ng/mL). WCEs were collected for IP with anti-SMAD3 antibody, followed by IB analysis. (**H**) HEK293T cells were transfected with vector, *EZH2*^WT^, or *EZH2*^Y641H^, and WCEs were collected for IP with anti-SMAD3 antibody, followed by IB analysis. All immunoblotting was performed 3 times, independently, with similar results.

The mass spectrometric results showed that the physical interaction between *EZH2* and *SMAD3* was dramatically enhanced by *TGFB1* stimulation (Supplemental Table 2). *EZH2* is a histone H3 methyltransferase capable of catalyzing the trimethylation of lysine 27 of histone H3 (*H3K27me3*) (37) and further confirmed the physical interaction between the *SMAD2*-*SMAD3* complex and *EZH2*, which could be significantly enhanced by *TGFB1* treatment (Figure 3, A and B, and Supplemental Figure 3, A–D). An in vitro GST pull-down assay further demonstrated the direct binding between *SMAD3* and *EZH2* (Supplemental Figure 3E). Next, we sought to determine whether *EZH2* could methylate *SMAD3*. Because the aa sequence of *SMAD3* is highly conserved with that of *SMAD2*, we transfected *Flag*-*EZH2* and *HA-SMAD2/3* into HEK293T cells and subsequently pulled down *HA-SMAD2*/*3*, using an anti–pan–K-me3 antibody for detection. Notably, we found that *EZH2* mediated the trimethylation of *SMAD3*, but not *SMAD2* (Supplemental Figure 3F). Remarkably, upon *TGFB1* treatment, the interaction between *EZH2* and *SMAD3* was enhanced, and *SMAD3* trimethylation was induced (Supplemental Figure 3G). *SMAD3* trimethylation and phosphorylation were enhanced in *EZH2*-overexpressing cells (Supplemental Figure 3H), while *SMAD3* trimethylation and phosphorylation were reduced in cells with *EZH2* knockdown or treated with *EZH2* inhibitors (GSK126 and GSK503; Figure 3C and Supplemental Figure 3I). Next, we tried to confirm whether *EZH2* triggered *SMAD3* trimethylation at K53/K333 and found that the double-methylation–deficient variant of *SMAD3* K53/333R abrogated the methylation of *SMAD3* upregulation induced by *EZH2*, while overexpression of *EZH2* did not affect other potential methylation sites, such as K81 and K117 (Figure 3D and Supplemental Figure 4A). Importantly, the in vitro methylation assay validated that *EZH2* could trimethylate *SMAD3* at the K53 and K333 sites (Figure 3E and Supplemental Figure 4B). In addition, we found that silencing of *EZH2* markedly inhibited *SMAD3* K53/K333 trimethylation (Figure 3F and Supplemental Figure 4C), whereas *EZH2* overexpression enhanced *SMAD3* K53/K333 trimethylation and *SMAD3*^S423/S425^ phosphorylation under *TGFB1* stimulation. Meanwhile, overexpression of WT *EZH2*, but not the *EZH2* H689A mutant deficient in methyltransferase activity (38, 39), enhanced basal and *TGFB1*-induced *SMAD3* at K53/K333 trimethylation and *SMAD3*^S423/S425^ phosphorylation (Figure 3G and Supplemental Figure 4D). Furthermore, the gain-of-methyltransferase function *EZH2* mutant *EZH2* Y641H (40) showed higher levels of *SMAD3* K53/K333 trimethylation compared with *EZH2* WT (Figure 3H). We found that P38 could be phosphorylated under *TGFB1* stimulation. Recent studies revealed that *P38*-mediated *EZH2* T372 phosphorylation induced its cytoplasmic localization to promote breast cancer metastasis (41, 42). Hence, we further investigated whether *EZH2* T372 phosphorylation was necessary for *TGFB1*-mediated, *EZH2*-catalyzed *SMAD3* K53/K333 methylation. Notably, we found that gain of WT *EZH2*, but not the *EZH2* T372A mutant, enhanced basal *TGFB1*-induced *SMAD3* at K53/K333 trimethylation and *SMAD3*^S423/S425^ phosphorylation, although cells ectopically expressing *EZH2* T372A had higher histone 3 at K27 trimethylation than did cells ectopically expressing WT *EZH2* (Supplemental Figure 4E). Collectively, our data suggest that *EZH2* triggered *SMAD3* trimethylation at the K53 and K333 sites. Therefore, *EZH2* is a *SMAD3* methyltransferase responsible for *SMAD3* K53/K333 trimethylation and *SMAD3*^S423/S425^ phosphorylation in response to *TGFB1* stimulation.

### EZH2-mediated tumor metastasis depends on SMAD3 methylation.

**Figure 4:**
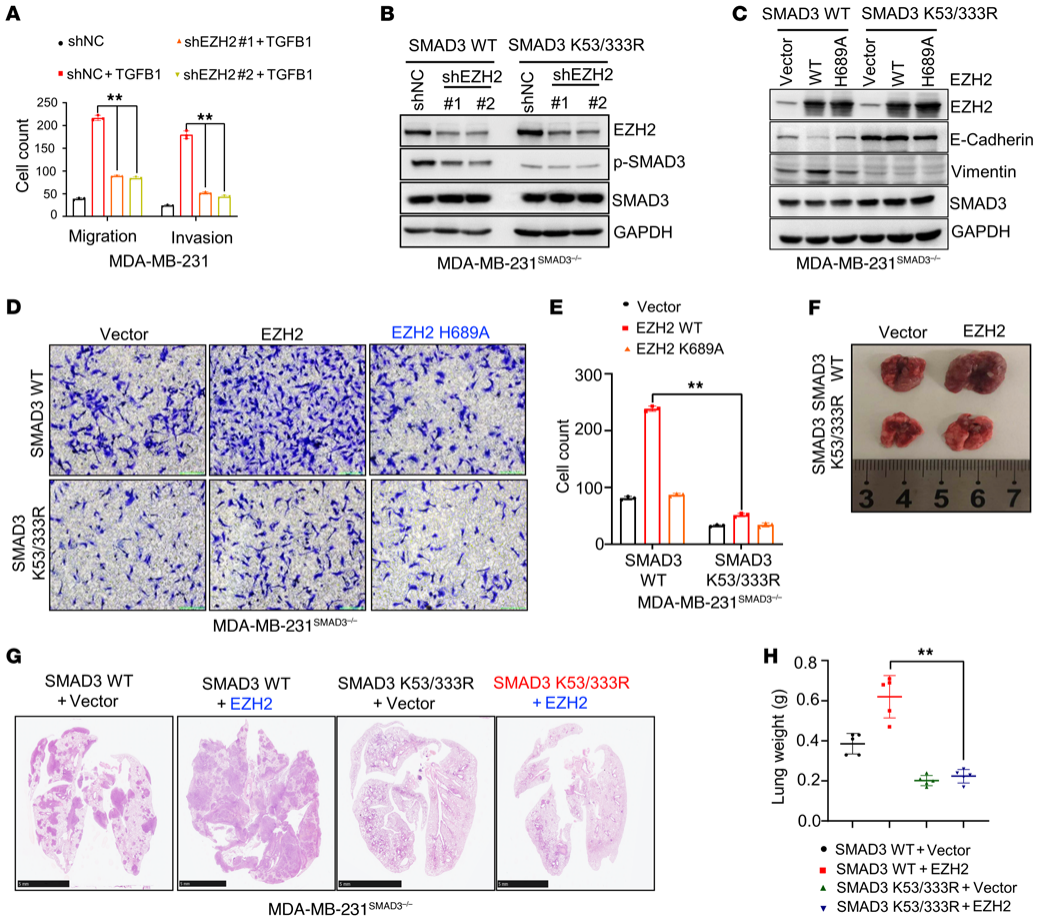
*EZH2* promotes cell migration and invasion dependent on methylation of *SMAD3* at K53 and K333. (**A**) Quantitative analysis of Transwell assay in the indicated MDA-MB-231 cells treated with *TGFB1* (5 ng/mL). (**B**) MDA-MB-231*^SMAD3–/–^* cells were stably transfected with WT *SMAD3* or *SMAD3* K53/333R plasmids and silenced with control or *EZH2* shRNA (nos. 1 and 2). WCEs were collected for IB analysis. (**C**) WT *Flag*-*EZH2* or a *Flag*-*EZH2* H689A plasmid was transfected into MDA-MB-231*^SMAD3–/–^* cells ectopically expressing WT *SMAD3* or *SMAD3* K53/333R, and WCEs were collected for IB analysis. (**D** and **E**) A Transwell cell invasion assay was performed using MDA-MB-231*^SMAD3–/–^* cells stably transfected with WT *SMAD3* or *SMAD3* K53/333R plasmids and transfected with a vector, *EZH2*^WT^, or *EZH2*^H689A^. Representative images (**D**) and quantitative analysis (**E**). Original magnification, ×200. (**F**–**H**). Representative lung image (**F**),H&E-stained lung sections (**G**), and scatter plot showing lung weights (**H**). Scale bars: 5 mm. All immunoblotting was performed 3 times, independently, with similar results. Data indicate the mean ± SD. **P < 0.05, by 2-tailed Student’s *t* test (**A**, **E**, and **H**).

*EZH2*, a histone H3 methyltransferase capable of catalyzing trimethylation on lysine 27 of histone H3 (*H3K27me3*), mediates the metastasis of various types of tumors (39, 43–45). In addition, the *TGFB* signaling pathway plays a crucial role in tumor metastasis (11, 36, 46). Signaling of both EZH2 and TGFB was found to be crucial for EMT of tumor cells and tumor metastasis. On the basis of these findings, we assumed that there is a relationship between *EZH2* and the *TGFB1* signaling pathway. We show in our study that the migratory and invasive abilities of cells induced by *TGFB1* could be inhibited by silencing *EZH2* (Figure 4A and Supplemental Figure 5, A–C). Moreover, knockdown of *EZH2* in *SMAD3* WT and *SMAD3* K53/333R cells resulted in decreased *SMAD3*^S423/S425^ phosphorylation in *SMAD3* WT cells, but not in *SMAD3* K53/333R–expressing cells, whose oncogenic capacity was lower than that of WT cells (Figure 4B), a finding that was consistent with the results seen with overexpression of *EZH2* in *SMAD3* WT and *SMAD3* K53/333R cells (Supplemental Figure 5D). Moreover, overexpression of *EZH2*, but not *EZH2* H689A, induced EMT in *SMAD3* WT cells, but not in *SMAD3* K53/333R or *SMAD3*-KO cells (Figure 4C and Supplemental Figure 5E). We also showed that *EZH2* promoted cell metastasis in *SMAD3* WT cells, but not in *SMAD3* K53/333R or *SMAD3*-KO cells, whereas the loss-of-function *EZH2* H689A mutant showed no such effect (Figure 4, D and E, and Supplemental Figure 5, F and G). A further in vivo xenograft study also showed that *EZH2* could significantly increase mouse lung metastatic nodule formation with WT *SMAD3*–expressing cells, but not with *SMAD3* K53/333R–expressing cells (Figure 4, F–H). Taken together, our results indicate that *EZH2* methylated *SMAD3* at K53 and K333, triggering tumor metastasis.

### SMAD3 K53/K333 methylation is necessary for SMAD3 membrane localization and phosphorylation.

**Figure 5:**
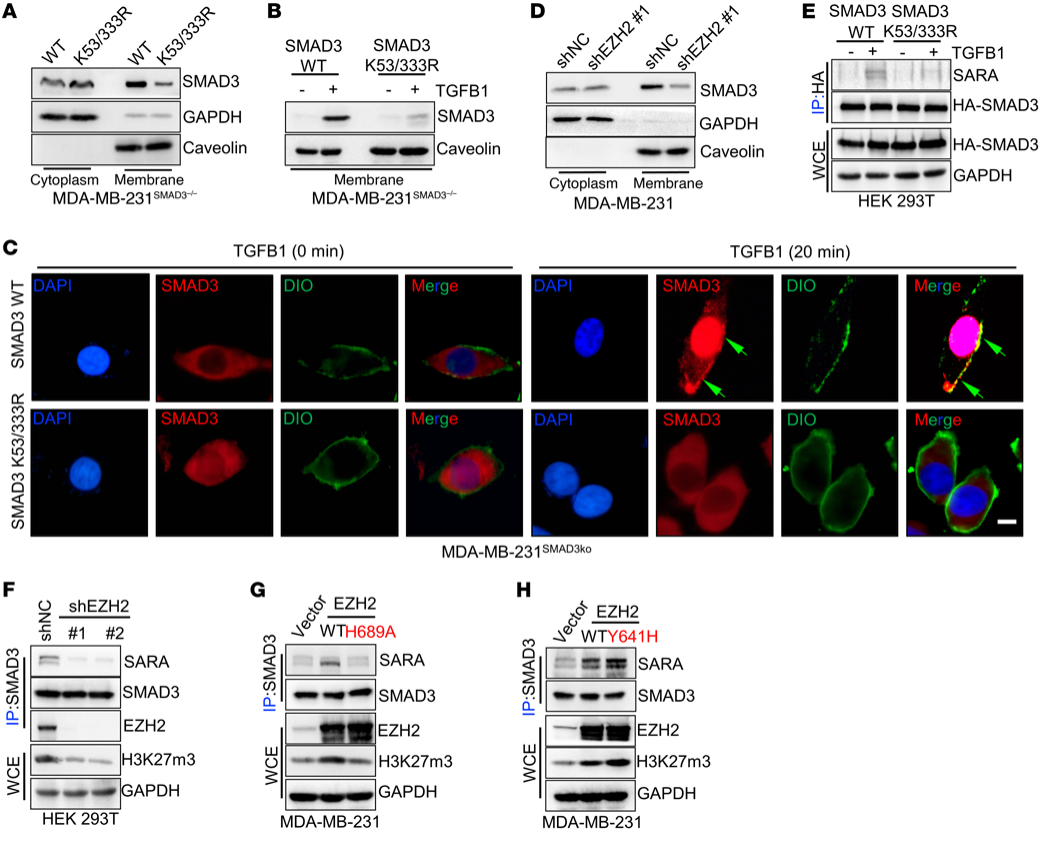
*SMAD3* K53/K333 methylation is essential for its membrane localization. (**A**) Membrane and cytosolic fractions from MDA-MB-231*^SMAD3–/–^* cells stably transfected with WT *SMAD3* or *SMAD3* K53/333R plasmids were collected and subjected to IB analysis. (**B**) Membrane fractions from MDA-MB-231*^SMAD3–/–^* cells stably transfected with WT *SMAD3* or *SMAD3* K53/333R plasmids and treated with *TGFB1* (5 ng/mL) were collected and subjected to IB analysis. (**C**) MDA-MB-231*^SMAD3–/–^* cells were stably transfected with WT *SMAD3* or *SMAD3* K53/333R plasmids and treated with *TGFB1* (5 ng/mL). IF images show the cellular localization of *SMAD3*. Scale bar: 10 μm. (**D**) Membrane and cytosolic fractions from MDA-MB-231 cells silenced with control or *EZH2* shRNA (no. 1) were collected and subjected to IB analysis. (**E**) HEK293T cells were transfected with WT HA-*SMAD3* or HA-*SMAD3* K53/333R plasmids and treated with *TGFB1* (5 ng/mL), and WCEs were collected for IP with anti-HA antibody, followed by IB analysis. (**F**) HEK293T cells were silenced with control or *EZH2* shRNA (nos. 1 and 2), and WCEs were collected for IP with anti-SMAD3 antibody, followed by IB analysis. (**G**) Co-IP of endogenous *SMAD3* from MDA-MB-231 cells transfected with *EZH2*^WT^ or *EZH2*^H689A^, followed by IB analysis. (**H**) Co-IP of endogenous *SMAD3* from MDA-MB-231 cells transfected with *EZH2*^WT^ or EHZ2^Y641H^, followed by IB analysis. All immunoblotting was performed 3 times, independently, with similar results.

Phosphorylation of *SMAD3* by *TGFB* type 1 receptors primarily occurs at the plasma membrane (17). To understand how *EZH2*-mediated *SMAD3* K53/K333 methylation regulates *SMAD3*^S423/S425^ phosphorylation and activation in response to *TGFB1*, we determined whether *EZH2*-mediated *SMAD3* K53/K333 methylation regulated *SMAD3* membrane recruitment upon *TGFB1* stimulation. Our data showed that *EZH2* deficiency or introduction of the *SMAD3* K53/333R mutation impaired basal and *TGFB1*-mediated *SMAD3* membrane recruitment (Figure 5, A–D, and Supplemental Figure 6, A–D). This indicates that *EZH2*-mediated *SMAD3* K53/K333 methylation regulates *SMAD3*^S423/S425^ phosphorylation by regulating *SMAD3* membrane recruitment.

*SMAD* anchor for receptor activation (*SARA*) belongs to a large family of proteins containing the *Fab1*, *YOTB*, *Vac1*, and *EEA1* protein (FYVE) domain, which confers the ability to interact with phosphatidylinositol 3-phosphate (PI3P), a phospholipid of membranes highly enriched in endosomes and directly involved in the recruitment of proteins (47). *SARA* contains structural motifs that interact with *SMAD2* and *SMAD3* (*SMAD2*/*3*), as well as a C-terminal region, all of which are required for interaction with the *TBRI*, turning on *TGFB* signaling by triggering *SMAD2*/3 membrane recruitment, phosphorylation, and nuclear translocation (35, 48, 49). We found that *EZH2*-mediated *SMAD3* K53/K333 methylation was essential for *SMAD3* membrane recruitment and phosphorylation at S423/S425 and that the activation of *TGFB* signaling was similar to that of *SARA*. We then studied the crosstalk between *SMAD3* K53/K333 methylation, as well as the interaction between *SARA* and *SMAD3*. Strikingly, the results showed that a methylation-deficient *SMAD3* K53/333R had an attenuated interaction with *SARA* compared with *SMAD3* WT, while silencing of *EZH2* had a similar result (Figure 5, E and F, and Supplemental Figure 6, E and F). Furthermore, overexpression of WT *EZH2*, but not *EZH2* H689A, increased the physical interaction between *SMAD3* and *SARA* (Figure 5G and Supplemental Figure 6G), whereas the *EZH2* Y641H mutant further enhanced the binding between *SMAD3* and *SARA* compared with WT *EZH2* (Figure 5H). Consistently, we found that silencing of *SARA* diminished *SMAD3*^S423/S425^ phosphorylation but not *SMAD3* methylation (Supplemental Figure 6H). Thus, we found that *EZH2*-mediated *SMAD3* K53/K333 methylation was essential for *SARA*-*SMAD3* interaction, which in turn mediated *SMAD3* membrane recruitment, phosphorylation at S423/S425, and activation of *TGFB* signaling.

### Inhibition of SMAD3 methylation with synthesized peptides blocks cancer cell metastasis.

**Figure 6:**
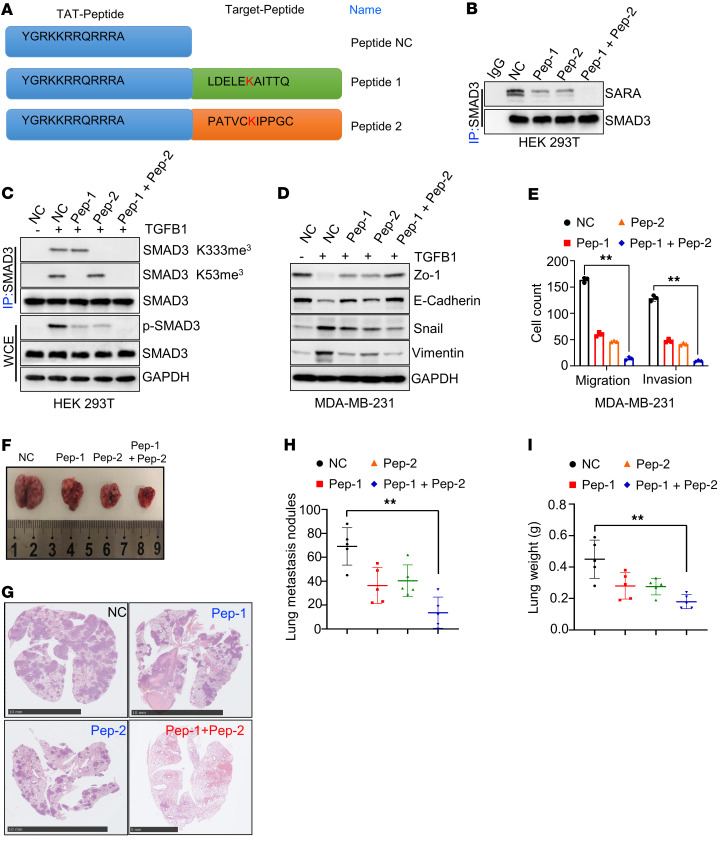
Targeting *SMAD3* K53/K333 methylation inhibits cancer metastasis. (**A**) The aa sequence of different TAT peptides. (**B** and **C**) HEK293T cells were treated with different TAT peptides (Pep-1, Pep-2) and *TGFB1* (5 ng/mL), and WCEs were collected for IP with anti-SMAD3 antibody, followed by IB analysis. (**D**) MDA-MB-231 were silenced with TAT peptides and treated with *TGFB1* (5 ng/mL), and WCEs were collected for IB analysis. (**E**) Quantitative analysis of Transwell cell migration and invasion assays using MDA-MB-231 cells treated with different TAT peptides. (**F** and **G**) Representative lung image (**F**) and H&E-stained lung sections (**G**). Scale bars: 10 mm. (**H** and **I**) Scatter plots show the number of lung metastatic nodes (**I**) and lung weights (**H**). All immunoblotting was performed 3 times, independently, with similar results. Data indicate the mean ± SD. **P < 0.05, by 2-tailed Student’s *t* test.

Given that *EZH2*-mediated *SMAD3* K53/K333 methylation is necessary for *TGFB*/*SMAD* signaling pathway activation and cancer metastasis, we investigated whether *SMAD3* methylation could be therapeutically targeted. To evaluate whether nonmethylated peptides containing methylation sites could inhibit endogenous *SMAD3* methylation and further restrain cancer metastasis, we first synthesized peptides that contained *SMAD3* methylation sites (48–58 aa for peptide 1 and 328–338 aa for peptide 2) with a *trans*-activator of transcription (TAT) tag placed in its N-terminal region (with a TAT domain as a negative control) (Figure 6A). To determine whether TAT peptides could restrain *SMAD3* methylation and phosphorylation, we first treated cells with TAT peptide 1 and/or TAT peptide 2. Interestingly, we found that TAT peptide 1 and TAT peptide 2 synergistically inhibited the interaction between *SMAD3* and *SARA* (Figure 6B). Importantly, TAT peptide 1 markedly inhibited *EZH2*/*TGFB1*-mediated *SMAD3* K53 methylation, but not *SMAD3* K333 methylation, and TAT peptide 2 could silence *EZH2*/*TGFB1*-mediated *SMAD3* K333 methylation, but not *SMAD3* K53 methylation (Figure 6C). Combining TAT peptides 1 and 2 markedly inhibited *EZH2*-mediated *SMAD3* K53/K333 methylation, as well as cancer cell EMT (Figure 6, C and D). In addition, immunofluorescence (IF) analysis showed that combining TAT peptides 1 and 2 markedly impaired *TGFB1*-mediated *SMAD3* membrane recruitment and nuclear localization (Supplemental Figure 7A). Transwell assays showed that the TAT peptides could inhibit cancer cell migratory and invasive abilities (Figure 6E and Supplemental Figure 7B). To test whether the TAT peptides inhibited breast cancer lung metastasis in vivo, we injected MDA-MB-231 cells into the tail vein of mice. Three days after injection, we injected nonmethylation TAT peptides or TAT control peptides (100 mL, 1 mg/mL per mouse) into the tail vein every 3 days. All mice were sacrificed 2 months later. The results showed that the lung metastatic nodules and lung weights in the nonmethylation TAT peptide–treated group were significantly decreased compared with those in the TAT control peptide–treated group, whereas peptides 1 and 2 had synergistic effects on the inhibition of tumor metastasis, indicating that nonmethylated peptides inhibited MDA-MB-231 lung colonization (Figure 6, F–I). Thus, targeting *SMAD3* methylation may be a potential therapeutic strategy to reverse the oncogenic processes.

### SMAD3 methylation is upregulated in breast cancer and associated with worse overall survival.

**Figure 7:**
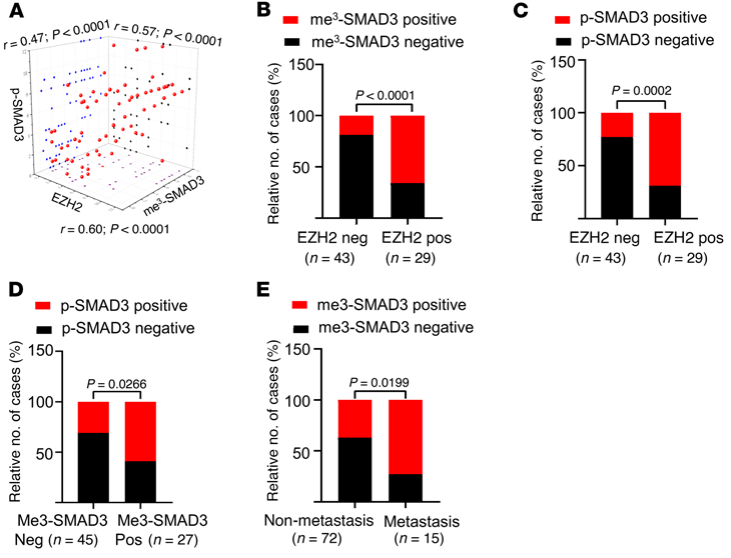
*SMAD3* methylation levels are positively correlated with *EZH2* and *SMAD3* phosphorylation levels. (**A**) Scatter plot of the IHC staining scores for *EZH2*, *SMAD3* K53/K333 trimethylation, and *SMAD3*^S423/S425^ phosphorylation in breast cancer (*n =* 75). All *P* and *r* values were calculated with Spearman’s *r* test. (**B**–**D**) Quantitative IHC staining scores showing the correlation between *EZH2*, *SMAD3* K53/K333 trimethylation, and *SMAD3*^S423/S425^ phosphorylation. neg, negat5ive; pos, positive. (**E**) Quantitative IHC staining score for staining of *SMAD3* K53/K333 trimethylation in nonmetastatic primary breast tumors and lung-metastatic primary breast tumors. *P =* 0.0199. Statistical significance was calculated with the χ^2^ test.

**Figure 8:**
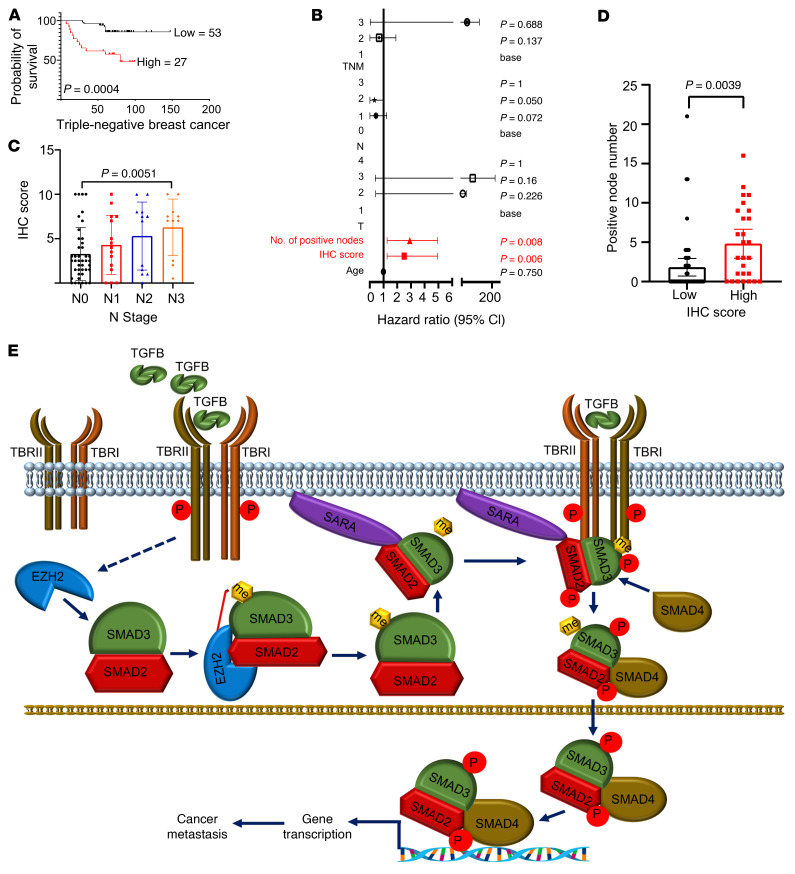
*SMAD3* K53/K333 methylation predicts poor survival of patients with cancer. (**A**) Kaplan-Meier survival analysis comparing high and low levels of *SMAD3* K53/K333 trimethylation using microarray results from a breast cancer specimen. *P =* 0.0004. (**B**) Cox regression analysis showing the significance of the relationship between *SMAD3* K53/K333 trimethylation expression and prognosis for patients with breast cancer in the presence of other clinical variables. (**C**) Scatter plot of *SMAD3* K53/K333 trimethylation IHC staining score comparing different N stages using microarray results from a breast cancer specimen (*n =* 80). *P =* 0.0051, by χ^2^ test. (**D**) Scatter plot of the number of cancer-positive lymph nodes comparing high and low levels of *SMAD3* K53/K333 trimethylation using microarray results from a breast cancer specimen (*n =* 80). *P =* 0.0039, by χ^2^ test. (**E**) Working model of *EZH2*-mediated *SMAD3* K53/K333 methylation crosstalk with TGFB-mediated *SMAD3* phosphorylation, membrane localization, and activation.

By examining the Gene Expression Profiling Interactive Analysis (GEPIA) databases, we found that *EZH2* was not only upregulated in diverse human cancers but also overexpressed in many different types of breast cancer (Supplemental Figure 6C). Using the Kaplan-Meier Plotter (KM Plotter) database (50), we found that higher *EZH2* expression was associated with poor survival outcomes for patients with breast cancer, as well as patients with breast cancer with lymph nodule metastasis (Supplemental Figure 8, A and B). To further investigate the relationship between *EZH2* and *SMAD3* methylation and phosphorylation in breast cancer, we performed IHC staining assays on breast carcinoma samples (Supplemental Figure 8C). *SMAD3* methylation IHC signal scores were calculated using the following formula: (K333me3 IHC signal score + K53me3 IHC signal score)/2. The results consistently showed that *EZH2* signals were positively correlated with *SMAD3* methylation and phosphorylation signals (Figure 7, A–C). In addition, *SMAD3* phosphorylation signals were also positively correlated with *SMAD3* methylation signals (Figure 7, A and D). Moreover, *SMAD3* methylation was highly expressed in the primary tumors of patients with metastatic breast cancer compared with expression in patients with nonmetastatic disease (Figure 7E and Supplemental Figure 8D). Next, we studied the relationship between *SMAD3* methylation expression levels and the overall survival of patients with cancer. We evaluated *SMAD3* methylation expression levels using microarray analysis of breast cancer specimens and found that *SMAD3* methylation levels were negatively correlated with patients’ overall survival, and the multivariate analysis showed that *SMAD3* methylation overexpression was an independent prognostic factor for patients with breast cancer (Figure 8, A and B, and Supplemental Figure 8E). Furthermore, we found that *SMAD3* methylation IHC scores were positively correlated with lymph nodule–positive numbers and N (node) stage (Figure 8, C and D, and Supplemental Figure 8F). Collectively, our data underscore the importance of *EZH2*-mediated *SMAD3* K53/K333 methylation in metastasis and indicate that *SMAD3* methylation might function as an important factor in predicting overall survival of patients with breast cancer.

## Discussion

Our data reveal insight into the regulation of *SMAD3* biological function. To determine whether *SMAD3* could be methylated, we first isolated *SMAD3* protein from cells and then incubated them with a pan–lysine trimethylation antibody or performed mass spectrometric analysis. We found that *SMAD3* K53 and K333 could be trimethylated and that only *SMAD3* trimethylation, but not dimethylation or monomethylation, was enhanced by *TGFB1* stimulation. Deletion of *SMAD3* methylation inhibited the interaction between *SMAD3* and its localization to cellular membrane *SARA*, decreases its C-terminal phosphorylation, and significantly repressed cancer cell EMT, colony formation ability, and metastasis, which phenocopied *SMAD3*-KO cancer cells.

When treated with *TGFB1*, the *SMAD2*-*SMAD3* complex could be respectively phosphorylated by the *TGFB1* receptor at the C-terminals of *SMAD2* and *SMAD3* (10, 17). Based on our observation of *SMAD3* methylation upregulated by *TGFB1* stimulation, we found that the interaction between *SMAD3* and *EZH2* could be dramatically enhanced by *TGFB1* treatment (Supplemental Table 2). Several studies showed that *TGFB1* could activate *P38* phosphorylation (10, 17, 41, 51). A recent study revealed that *P38*-mediated *EZH2* phosphorylation induced its cytoplasmic localization to promote breast cancer metastasis (42). In our study, we identified *SMAD3* as a substrate of *EZH2* and showed that *EZH2*-mediated *SMAD3* K53/K333 methylation crosstalks with *TGFB1*-mediated *SMAD3* C-terminal phosphorylation. We believe this study expands our understanding of *TGFB1*/*EZH2*-mediated tumor metastasis.

*EZH2* is amplified and overexpressed and is associated with poor survival in various cancers (52–54). Recent studies have identified *EZH2* as a potential target for cancer treatment (55, 56). Consistently, we found that *EZH2*-mediated *SMAD3* K53/K333 methylation was clearly upregulated in metastatic breast cancer and correlated with *SMAD3* C-terminal phosphorylation. Furthermore, we found that *SMAD3* K53/K333 methylation levels were highly consistent and associated with poor survival in breast cancer. Targeting *SMAD3* methylation using TAT peptides 1 and 2 dramatically inhibited breast cancer metastasis (Figure 6). The mostly tumorigenesis function of *EZH2* was previously thought to be dependent on *EZH2*-induced histone *H3K27me3* modification. However, we found that *EZH2*-mediated tumor metastasis markedly abrogated the expression of *SMAD3* K53/333R methylation–deficient mutant cells compared with that of WT *SMAD3* cells. These results showed that *EZH2*-mediated biological function was partly dependent *EZH2*-mediated *SMAD3* K53/K333 methylation.

Pharmacological targeting of *SMAD3* activity, such as with SIS3 (a specific inhibitor of *SMAD3* C-terminal phosphorylation), was proposed as a potential therapeutic strategy to treat various diseases, including metastatic cancer (57). Suppression of *SMAD3* activity was shown to depress fibrosis, apoptosis, and inflammation in mouse kidneys with unilateral ureteral obstruction and inhibited the development of diabetic nephropathy in a mouse model of type 1 diabetes (58, 59). In an in vitro experiment, inhibition of *SMAD3* C-terminal phosphorylation reversed *ABCB1*- and *ABCG2*-mediated multidrug resistance in cancer cell lines, *E4BP4*-mediated NK cell development, and tumor metastasis (57, 60, 61). In clinical experiments, *EZH2* inhibitors were shown to impede the progression of many cancer types, including lymphoma and solid tumors (62, 63). We observed that when *SMAD3* was in a hypermethylated state, the cancer cells were prone to EMT. We also showed that patients with breast cancer who had lung or lymphatic metastases had higher *SMAD3* methylation levels. In our study, we introduced a therapeutic drug to target *SMAD3* K53/K333 methylation. We found that TAT peptides inhibited not only *TGFB*/*SMAD* signaling pathway but the *EZH2* signaling pathway as well. Importantly, although *EZH2* inhibitors could be used to block *EZH2*/*SMAD3* signaling, their side effects may limit their applications because of the inhibition of *H3K27* trimethylation. Furthermore, other nonhistone substrates have also been identified, such as EZH2 catalysis of *STAT3* methylation, which is essential for *STAT3* transcriptional activation (38, 39), and *SYMD2* stabilization, which was found to be regulated by *EZH2*-mediated *SYMD2* methylation (43). In our study, the *EZH2* inhibitors also had inhibitory roles in *STAT3* and *SYMD*2 signaling. However, unlike *EZH2* inhibitors that block *H3K27* trimethylation, the targeting of TAT peptides was more specific, and the potential side effects might be more manageable.

In summary, we identified a PTM of *SMAD3*, methylation. We showed that *SMAD3* acted as a substrate for *EZH2* and that *EZH2*-mediated *SMAD3* K53/K333 methylation was essential for *TGFB1*-mediated *SMAD3* recruitment to its cellular membrane location, *SARA*, and for C-terminal phosphorylation. Importantly, *EZH2*-mediated *SMAD3* methylation not only rendered cancer cells more vulnerable to *TGFB1* and promoted cancer cell EMT and metastasis, but also showed a positive correlation with poor patient survival (Figure 8E). Moreover, pharmacological inhibition of *SMAD3* methylation dramatically inhibited cancer metastasis. We believe this study expands our understanding of *TGFB1*/*EZH2*-mediated tumor metastasis. Once *EZH2* is amplified in cancer cells, *TGFB1*-mediated gene expression might be more vulnerable to activation and further facilitate tumor progression. Overall, *TGFB1*-mediated, *EZH2*-catalyzed *SMAD3* K53/K333 methylation could function as a predictive marker of survival for patients with cancer and potentially serve as a therapeutic target for patients with metastatic cancer.

## Methods

### Cell lines and cell culturing.

Human MDA-MB-231, MCF-7, and HEK293T cells were purchased from the American Type Culture Collection (ATCC). MDA-MB-231 cells were maintained in L-15 medium (HyClone, GE Healthcare). MCF-7 and HEK293T cells were cultured in DMEM basal medium. FBS (10%, v/v; Gibco, Thermo Fisher Scientific) was added to the DMEM and L-15 basal culture medium. MDA-MB-231 cells were cultured at 37°C in a 100% air incubator, and other cells were cultured at 37°C in a 5% (v/v) CO_2_ incubator.

### Antibodies and chemicals.

The following commercially available primary antibodies were used: anti-EZH2 (A11085, A13867, and A19577, ABclonal); anti–Flag tag (AE063, ABclonal and 14793S, Cell Signaling Technology); anti–HA tag (AE036, ABclonal and 3724T, Cell Signaling Technology); anti-SMAD3 (A19115, ABclonal and sc-101154, Santa Cruz Biotechnology); anti–p-SMAD3^S423/S425^ (AP0727, ABclonal); anti-SMAD2 (A19114, ABclonal); anti-GAPDH (sc-47724, Santa Cruz Biotechnology); anti-vimentin (5741, Cell Signaling Technology); anti–E-cadherin (610182, BD Transduction Laboratories); anti–ZO-1 (sc-33725, Santa Cruz Biotechnology); anti-Snail (3879, Cell Signaling Technology); anti–pan–monomethyl lysine (A18293, ABclonal); anti–pan–dimethyl lysine (14117, Cell Signaling Technology); anti–pan–trimethyl lysine (14680, Cell Signaling Technology); anti–trimethyl histone H3-K27 (A2363, ABclonal); anti–histone H3 (4499, Cell Signaling Technology); anti–caveolin-1 (A19006, ABclonal); anti-SARA (A16465, ABclonal); anti-HGS (A1970, ABclonal); and anti-DAB2 (A10349, ABclonal). The *SMAD3* K53/K333 trimethylation rabbit polyclonal antibody was prepared by ABclonal (https://abclonal.com.cn). The following secondary antibodies were used in the immunofluorescence assays: anti–Dylight 549, goat anti–rabbit IgG (A23320, Abbkine); anti–Dylight 488, goat anti–mouse IgG (A23210, Abbkine); anti–Dylight 488, goat anti–rabbit IgG (A23220, Abbkine); and anti–Dylight 549, goat anti–mouse IgG (A23310, Abbkine). The following other small-molecule materials were used: GSK126 (T2079, Topscience); GSK503 (T1775,Topscience); *TGFB1* (HY-P7118, MedChemExpress); and DiO (C1038, Beyotime).

### Plasmid construction.

The expression vectors encoding pcDNA3.1-*HA*-*SMAD3*, pcDNA3.1-*HA*-*SMAD2*, and pcDNA3.1-*Flag*-*EZH2* were purchased from AuGCT (http://www.augct.com). pLKO-AS3W-*HA*-*SMAD3* and pLKO-AS3W-*Flag*-*EZH2* plasmids were constructed by inserting the indicated DNAs into the pLKO-AS3W vector. The *SMAD3* and *EZH2* mutants were generated using a Mut Express II Fast Mutagenesis Kit V2 (C214-01, Vazyme). pLKO.1-lentiviral vectors expressing *EZH2* shRNA were based on the following sequences: 5′-AAGAGGTTCAGACGAGCTGAT-3′ and 5′-GCTAGGTTAATTGGGACCAAA-3′.

### Construction of cell lines.

We used pLKO.1-sh*EZH2*, MD2-G, and the PPAX 3-pack system for the expression-silencing virus. We used the pLKO-AS3W–indicated genes, MD2-G, and the PPAX 3-pack system to generate high-expression viruses. *SMAD3*-KO virus was purchased from Genechem Shanghai. The sequences of the sgRNAs against the *SMAD3*-KO virus were as follows: 5′-GTGGTTCATCTGGTGGTCAC-3′; 5′-GCCGGCTCGCAGTAGGTAAC-3′ and 5′-ATTCGGAGCGCTTCTGCCTA-3′. All viruses were respectively transfected into the identified cells. After 12 hours, the medium was substituted with fresh complete medium. After 48 hours, 1 μg/mL puromycin was used to select the infected cells.

### Mass spectrometric analyses.

Briefly, for protein elution, denaturation, reduction, and alkylation, beads samples obtained from the co-IP experiment were incubated with the reaction buffer containing sodium dehydrocholate (SDC), Tris (2 carboxyethyl) phosphine (TCEP), and chloroacetamide (CAA) at 95°C for 10 minutes. The supernatant was diluted 2 times with H_2_O. Trypsin (1 μg) was added for overnight digestion at 37°C. The next day, peptide purification was performed using SDB desalting columns. The peptide eluate was vacuum dried and stored at –20 °C for later use. Liquid chromatography tandem mass spectrometry (LC-MS/MS) data acquisition was carried out on a Q Exactive Plus LC-MS/MS mass spectrometer equipped with a nanospray source (Thermo Fisher Scientific). Peptides were dissolved in MS loading buffer (0.1% formic acid), loaded onto a C18 trap column through an autosampler, and then eluted into a C18 analytical column (50 μm × 150 mm, 2 μm particle size, 100 Å pore size). Mobile phase A (0.1% formic acid) and mobile phase B (90% ACN, 0.1% formic acid) were used to establish a 60-minute separation gradient. A constant flow rate was set at 300 nL/min. Data were acquired using a spray voltage of 2 kV, an ion funnel radio frequency (RF) of 40, and ion transfer tube temperature of 320°C. For data-dependent acquisition (DDA) mode analysis, each scan cycle consisted of 1 full-scan mass spectrum (resolution 70 K, scan range: 350–1800 *m/z*; automatic gain control [AGC]: 3 × 10^6^; ion trap [IT]: 20 ms) followed by 15 MS/MS events (resolution 17.5 K; AGC: 2 × 10^5^; IT: 50 ms). High-energy collision dissociation (HCD) collision energy was set at 28. The isolation window was set at 1.6 Da. The dynamic exclusion time was set at 35 seconds. MS raw data were analyzed with MaxQuant software (version 1.6.6) using the Andromeda database search algorithm and the MaxLFQ function. Spectra files were searched using the default parameters, except for label-free quantification mode, the minimum ratio count was set at 1, and the match-between-runs function was checked. The search results were filtered with a 1% FDR at both protein and peptide levels.

### Cytosolic and membrane fractions.

Cytosolic and membrane fractions of the identified cells were isolated using a membrane protein extraction kit (C500049, Sangon Biotech) according to the manufacturer’s instructions.

### Western blot and IP assays.

Cells were washed in cold PBS 3 times and lysed with NP-40 buffer for 30 minutes at 4°C. The protein concentration was measured with a Bicinchoninic Acid Assay (BCA) kit (Thermo Fisher Scientific). Membrane and cytosol fractions were isolated according to the manufacturer’s instructions (15F17B55, Boster Biological Technology). Next, 5× protein sample buffer (250 mM Tris–HCl [pH 6.8], 10% SDS, 30% glycerol, 5% β-mercaptoethanol, bromophenol blue) was added, followed by boiling of the samples at 95°C for 10 minutes. Proteins were separated by electrophoresis in an 8%~12% premade sodium dodecylsulfate-polyacrylamide minigel and then transferred onto a PVDF membrane. The membranes were incubated with the indicated antibodies overnight at 4°C, followed by incubation with HRP-conjugated secondary antibodies for 2 hours at room temperature. Immunoreactive bands were detected by chemiluminescence. For IP, the indicated primary antibodies were incubated with magnetic beads (HY-K0205, MedChemExpress [MCE]) overnight at 4°C and subsequently rotated with cell lysis, followed by Western blot analysis.

### In vitro methylation assays and in vitro binding assays.

In vitro methylation assays were performed as described previously (64). Briefly, EZH2 (ab132934, Abcam) and SMAD3 (ab151882, Abcam) proteins were purchased. *Flag*-tagged EZH2 protein was expressed and purified from HEK293T cells. Synthesized SMAD3 peptides from Abclone served as substrates. In each tube contained 1 μg Flag-tagged EZH2, 5 μL 5× protein lysine methyltransferase (PKMT) buffer (10 mM Tris–HCl [pH 8], 2% glycerol, 0.8 mM KCl, 1 mM MgCl_2_), 13 μM S-adenosyl-l-methionine (SAM), and GST-SMAD3–purified protein or SMAD3 peptides as substrates, with addition of H_2_O for a final volume of 25 μL. The reaction tubes were incubated at 37°C for 10 hours. The reaction was stopped by adding 5× protein sample buffer (250 mM Tris-HCl [pH 6.8], 10% SDS, 30% glycerol, 5% β-mercaptoethanol, bromophenol blue) followed by boiling of the samples at 95°C for 10 minutes. For the in vitro binding assays, purified *EZH2* protein was incubated with GST or GST-SMAD3 fusion proteins bound to glutathione sepharose beads at 4°C overnight followed by Western blot analysis.

### IHC analyses.

All clinical samples were obtained from patients with breast cancer who underwent surgical resection at Wuhan Tongji Hospital. None of the patients received chemotherapy or radiation therapy before surgery. For ICH analyses, tissue specimens were first cut into sections of 4 μm thickness and then fixed with 4% paraformaldehyde for 15 minutes at room temperature. The samples were stained with the indicated primary antibodies overnight at 4°C, followed by staining with secondary antibodies at room temperature for 1 hour. Two experienced pathologists received and scored the immunostaining results independently. The immunoreactive score (IRS) was used to quantify the IHC results. The percentage of positively stained tumor cells was scored as follows: 1 (<10%), 2 (10%–50%), 3 (50–75%), and 4 (>75%). Staining intensity was scored as follows: 0 = no staining; 1 = weak staining; 2 = moderate staining; 3 = strong staining. Finally, the IHC score was calculated by multiplying the score of the percentage of positively stained tumor cells and staining intensity, which ranged from 0 to 12.

### Immunocytochemistry.

For immunocytochemistry, the indicated cells were fixed with 4% paraformaldehyde for 15 minutes at room temperature. Next, the cells were permeabilized in 5% Triton X-100 for 5 minutes and then cultured with primary antibodies for 1 hour at room temperature. Anti–mouse Alexa Fluor 488 or 594 dye–conjugated and/or anti–rabbit Alexa Fluor 488 or 594 dye–conjugated secondary antibodies were used. Cell nuclei were stained with DAPI. Then, the cells were imaged using a multiphoton confocal laser scanning or fluorescence microscope.

### Transwell assays.

For Transwell migration and invasion assays, 4 × 10^4^ cells (for invasion) or 10 × 10^4^ cells (for migration) suspended in medium without FBS were seeded in the upper chamber membranes with or without Matrigel (BD Biosciences), and 500 μL complete medium was added to the lower chamber. After 6–12 hours, the cells were fixed with 4% paraformaldehyde for 15 minutes at room temperature and then stained with 0.1% Crystal Violet. Cells of the inner chamber were wiped out with a cotton swab. Cell numbers were calculated with a microscope.

### Mouse xenograft assays.

Four-week-old female BALB/c nude mice were purchased from Beijing Huafukang Bioscience. The indicated cancer cells were collected and washed twice with PBS. Cells were resuspended with PBS and injected into the tail vein (4 × 10^5^ cells per mouse). TAT control and TAT nonmethyl peptides (100 μL, 1 μg/mL) were injected into the tail vein at the indicated time intervals. After 2 months, the mice were sacrificed and their lungs isolated and imaged.

### Statistics.

All statistical analysis was conducted using GraphPad Prism 8.0 (GraphPad Software) and SPSS 22.0 (IBM). All data represent the mean ± SD. Spearman’s *r* was calculated for ordinal data. Fisher’s exact test was used in the contingency tables. Pearson’s χ^2^ and Mann-Whitney *U* tests were used to assess the correction for between levels of protein expression. The difference was tested using a 2-tailed Student’s *t* test or 1-way ANOVA. A *P* value of less than 0.05 was considered statistically significant.

### Study approval.

This study was approved by the Huazhong University of Science and Technology Ethics Committee. Written informed consent was obtained from all patients in this study. All mouse experiments were approved by the IACUC of Tongji Hospital.

## Author contributions

CH and Fuqing Hu performed the majority of the experiments. CH and GW wrote the manuscript. CH, DS, AL, QW, Xiaowei She, GW, and JH designed the research studies and acquired data. YC, Fayong Hu, and FX helped analyze data. XL and YF provided reagents. LC and XS acquired data. XY provided advice for our study. All authors approved this study.

## Supplementary Material

Supplemental data

Supplemental table 1

Supplemental table 2

## Version 1 01/27/2022

In-Press Preview

## Version 2 03/01/2022

Electronic publication
