# Supplementary material for: EZH2-triggered methylation of SMAD3 promotes its activation and tumor metastasis
Source: J Clin Invest. 2022 Mar 1;132(5):e152394. doi: 10.1172/JCI152394 (PMC8884901; doi:10.1172/JCI152394)
Supplement: Supplemental data [file jci-132-152394-s210.pdf]

Figure S1. Identified *SMAD3* methylation site by Mass spectrum (A) whole cell extracts (WCE) of HEK293T cells were collected for immunoprecipitation (IP) with *SMAD3* antibody, followed by Immunoblots (IB) analysis. **(B)** HEK293T cells were serum-starved, treated with *TGFB1*(5ng/ml) for indicated time points, and whole cell extracts (WCE) were collected for immunoprecipitation (IP) with *SMAD3* antibody, followed by Immunoblots (IB) analysis. **(C, D)** HEK293T cells transfected with *HA-SMAD3* and treated with *TGFB1*(5ng/ml), WCE were collected for IP with *HA* antibody, followed by IB analysis. **(E)** HEK293T cells were serum-starved, treated with *TGFB1*(5ng/ml) and Dznep, WCE were collected for IP with *SMAD3* antibody, followed by IB analysis. **(F)** Showing results from mass spectrometry analysis of *SMAD3* potential methylation sites. **(G)** HEK293T cells transfected with *HA-SMAD3* WT or K53/333R mutant plasmids, WCE were collected for IB analysis. **(H)** IB analysis of *SMAD3* expression level in *SMAD3* WT and *SMAD3* KO MDA-MB-231 cells. **(I)** IB analysis of *SMAD3* expression level in MDA-MB-231$^{SMAD3-/-}$ cell expressing ectopic *SMAD3* WT or *SMAD3* K53/333R. All immunoblots are performed three times, independently, with similar results.

Figure S2. The biological function of *SMAD3* K53/333R cells phenocopied *SMAD3* knock out cells. (A) MDA-MB-231 cells transfected with *HA-SMAD3* WT or K53/333R mutant plasmids and treated with *TGFB1*(5ng/ml), quantitative RT-PCR analysis of *TGFB*/*SMAD3* signaling pathway downstream genes expression, including *CTGF*, *PAI1*, *PDGFB*, and *SMAD7*; **p<0.05. **(B)** MCF-7 cells transfected with *HA-SMAD3* WT or K53/333R mutant plasmids and treated with *TGFB1*(5ng/ml), quantitative RT-PCR analysis of *TGFB*/*SMAD3* signaling pathway downstream genes expression, including *CTGF*, *PAI1*, *P21*, and *SMAD7*; **p<0.05. **(C)** Transwell cell migration and invasion assay in MDA-MB-231$^{SMAD3-/-}$ cells stably transfected with *SMAD3* WT or *SMAD3* K53/333R plasmids. **(D)** Immunofluorescence (IF) analysis of EMT marks in indicated cells; Scale bar, 50um. **(E, F)**. Representative lung image **(E)** and H&E-stained lung sections **(F)**. **(G, H)** Scatter plot showing lung metastatic nodules **(G)** and lung weight **(H)**; **p<0.05. **(I)** Representative image of IHC staining for *SMAD3* K53 and K333 tri-methylation and *SMAD3* S423/S425 phosphorylation for

lung sections; scale bar, 100um. All immunoblots are performed three times, independently, with similar results. Error bars are mean ± s.d. Statistical significance was assessed using Student's two-tailed t-test.

Figure S3. *EZH2* triggers *SMAD3* methylation. (A) HEK293T cells transfected with *HA-SMAD3* or *Flag-EZH2* plasmids, WCE were collected for IP with *HA* or *Flag* antibody, followed by IB analysis. **(B, C)** WCE from MDA-MB-231 **(B)** and MCF-7 **(c)** cells were collected for IP with *EZH2* antibody, followed by IB analysis. **(D)** MDA-MB-231 cells were serum-starved, treated with *TGFB1*(5ng/ml), and WCE were collected for IP with *SMAD3* antibody, followed by IB analysis. **(E)** *EZH2* protein was incubated with GST-*SMAD3* WT or GST protein for the *in vitro* binding assay, followed by immunoprecipitation (IP) of GST-*SMAD3* and IB analysis. **(F)** HEK293T transfected with *HA-SMAD2* or *HA-SMAD3* and *Flag-EZH2*, WCE were collected for IP with HA antibody, followed by IB analysis. **(G)** HEK293T transfected with *HA-SMAD3* and *Flag-EZH2*, then treated with *TGFB1*(5ng/ml), WCE were collected for IP with *HA* antibody, followed by IB analysis. **(H)** MCF-7 cells transfected with *HA-SMAD3* and *Flag-EZH2*, then treated with *TGFB1*(5ng/ml), WCE were collected for IP with *HA* antibody, followed by IB analysis. **(I)** HEK293T (left) and MCF-7 (right) cells transfected with *HA-SMAD3* and silenced with control (ShNC) or *EZH2* ShRNA (#1 and #2), then treated with *TGFB1*(5ng/ml), WCE were collected for IP with *HA* antibody, followed by IB analysis. All immunoblots are performed three times, independently, with similar results.

Figure S4. *EZH2* triggers *SMAD3* methylation at K53 and K333. (A) HEK293T cells transfected with *HA-SMAD3* WT or mutant plasmids and *Flag-EZH2* plasmid as indicated, WCE were collected for IP with *HA* antibody, followed by IB analysis. **(B)** Conducting in vitro methylation assays using purified *EZH2* protein as the source of methyltransferase, and the synthetic *SMAD3* non methylation peptides containing K53 or K333 as the substrate. *SMAD3* K53 tri-methylation peptides (left) and *SMAD3* K333 tri-methylation peptides (right) as a positive control. **(C)** MCF-7 silenced with control (ShNC) or *EZH2* ShRNA (#1 and #2), WCE were collected for IP with *SMAD3* antibody, followed by IB analysis. **(D)** MDA-MB-231(left) and MCF-7(right) cells transfected

with Vector, *EZH2*^{WT} and treated with *TGFB1*(5ng/ml), WCE were collected for IP with *SMAD3* antibody, followed by IB analysis. **(E)** HEK 293T cells transfected with Vector, *EZH2*^{WT}, *EZH2*^{T372A}, WCE were collected for IP with *SMAD3* antibody, followed by IB analysis. All immunoblots are performed three times, independently, with similar results.

Figure S5. *EZH2*-mediated tumor metastasis depended on SMAD3 methylation. (A, B) Trans-well cell migration assay in MCF-7 silenced with control (ShNC) or *EZH2* ShRNA (#1 and #2) **(A)**, quantitative cell migration number was shown **(B)**. **(C)** Trans-well cell migration and invasion assay in MDA-MB-231 cells silenced with control (ShNC) or *EZH2* ShRNA (#1 and #2). **(D)** *Flag-EZH2* plasmid was transfected into MDA-MB-231$^{SMAD3-/-}$ cell ectopic expressing *SMAD3* WT or *SMAD3* K53/333R, followed by IB analysis. **(E)** *Flag-EZH2* WT or *Flag-EZH2* H689A plasmid transfected into MDA-MB-231 KO or MDA-MB-231 WT cells, WCE was collected for IB analysis. **(F, G)** Trans-well cell migration assay in MDA-MB-231 KO or MDA-MB-231 WT cells**(F)**, quantitative cell migration number was shown **(G)**; **p<0.05. All immunoblots are performed three times, independently, with similar results. Error bars are mean ± s.d. Statistical significance was assessed using Student's two-tailed t-test.

Figure S6. Deletion of SMAD3 K53 and K333 methylation inhibited its membrane localization. (A, B) IB analysis of *EZH2* expression level in MDA-MB-231**(A)** and HEK293T**(B)** cells silenced with control (ShNC) or *EZH2* ShRNA (#1). **(C)** HEK293T cells silenced with control (ShNC) or *EZH2* ShRNA (#1), cellular membrane was extracted, followed by IB analysis of *SMAD3* expression level. **(D)** MAD-MB-231 cells silenced with control (ShNC) or *EZH2* ShRNA (#1) and treated with *TGFB1*(5ng/ml), immunocytochemistry analysis of the cellular localization of *SMAD3*. **(E)** WCE from MDA-MB-231 cells were collected for IP with *SMAD3* antibody, followed by IB analysis. **(F)** WCE from MDA-MB-231 $^{SMAD3-/-}$ cell ectopic expressing *SMAD3* WT or *SMAD3* K53/333R were collected for IP with *SMAD3* antibody, followed by IB analysis. **(G)** HEK293T cell transfected with *Flag-EZH2* plasmid, WCE was collected for IP with *SMAD3* antibody, followed by IB analysis. **(H)** HEK293T cells silenced with control (ShNC) or *EZH2* ShRNA (#1 and #2), WCE were collected for IP with *SMAD3*

antibody, followed by IB analysis. All immunoblots and immunocytochemistry assays are performed three times, independently, with similar results.

Figure S7. *EZH2* expression level is upregulated in various kinds of cancer and has a negative cancer patients' survival time. (A) MDA-MB-231 cells treated with *TGFB1*(5ng/ml) with or without TAT-peptides, IF showing the cellular localization of *SMAD3*. **(B)** Trans-well cell migration assay in MDA-MB-231 treated with TAT-peptides. **(C)** Analysis of *EZH2* expression level in various kinds of cancer using GEPIA database.

Figure S8. *EZH2* expression level has a positive correlation with *SMAD3* K53/K333 methylation level and SMAD3 S423/S425 phosphorylation level in breast cancer. (**A, B)** Analysis of patients' survival time in breast cancer patients**(A)** or breast patients with lymph node positive**(B)** grouped by *EZH2* expression level using GEPIA database. **(C)** Representative image of IHC staining for *EZH2*, *SMAD3* K53 and K333 tri-methylation and *SMAD3* S423/S425 phosphorylation in breast cancer; scale bar, 100um. **(D)** Representative image of IHC staining of *SMAD3* K53 and K333 tri-methylation in non-metastasis primary breast tumors and lung-metastasis primary breast tumors. **(E, F)** Representative image **(E)** of IHC staining of *SMAD3* K53 and K333 tri-methylation in breast cancer, quantitative IHC staining score was shown **(F)**; p=0.0018. Statistical significance is assessed using Chi-Square test.

Figure.S1

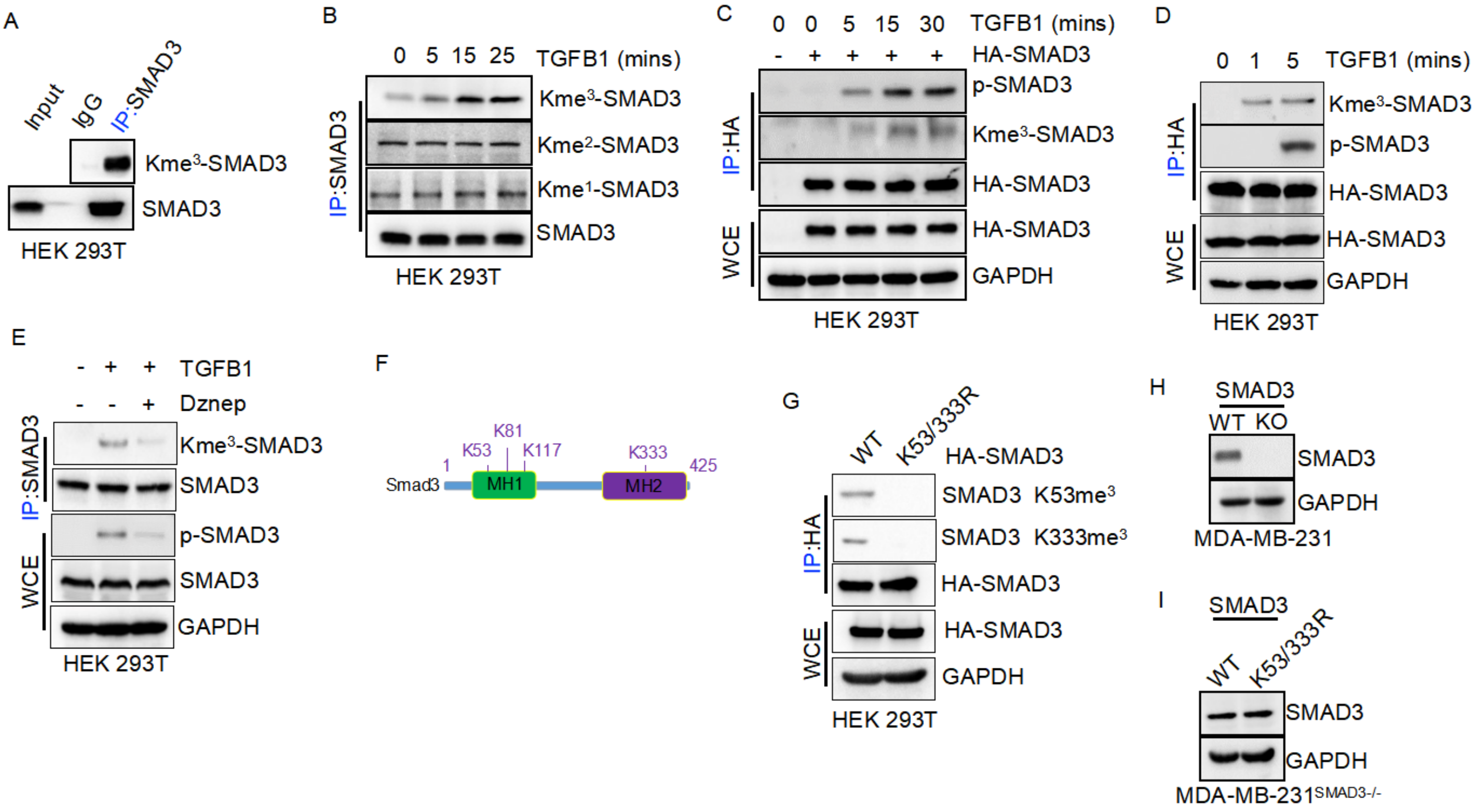
A
Input
IgG
IP:SMAD3
Kme3-SMAD3
SMAD3
HEK 293T
B
0 5 15 25 TGFB1 (mins)
IP:SMAD3
Kme3-SMAD3
Kme2-SMAD3
Kme1-SMAD3
SMAD3
HEK 293T
C
0 0 5 15 30 TGFB1 (mins)
- + + + + HA-SMAD3
IP:HA
p-SMAD3
Kme3-SMAD3
HA-SMAD3
WCE
HA-SMAD3
GAPDH
HEK 293T
D
0 1 5 TGFB1 (mins)
IP:HA
Kme3-SMAD3
p-SMAD3
HA-SMAD3
WCE
HA-SMAD3
GAPDH
HEK 293T
E
- + + TGFB1
- - + Dznep
IP:SMAD3
Kme3-SMAD3
SMAD3
WCE
p-SMAD3
SMAD3
GAPDH
HEK 293T
F
Smad3
1
K53
K81
K117
MH1
K333
MH2
425
G
WT
K53/333R
HA-SMAD3
IP:HA
SMAD3 K53me3
SMAD3 K333me3
HA-SMAD3
WCE
HA-SMAD3
GAPDH
HEK 293T
H
SMAD3
WT KO
SMAD3
GAPDH
MDA-MB-231
I
SMAD3
WT
K53/333R
SMAD3
GAPDH
MDA-MB-231SMAD3-/-

Figure.S2

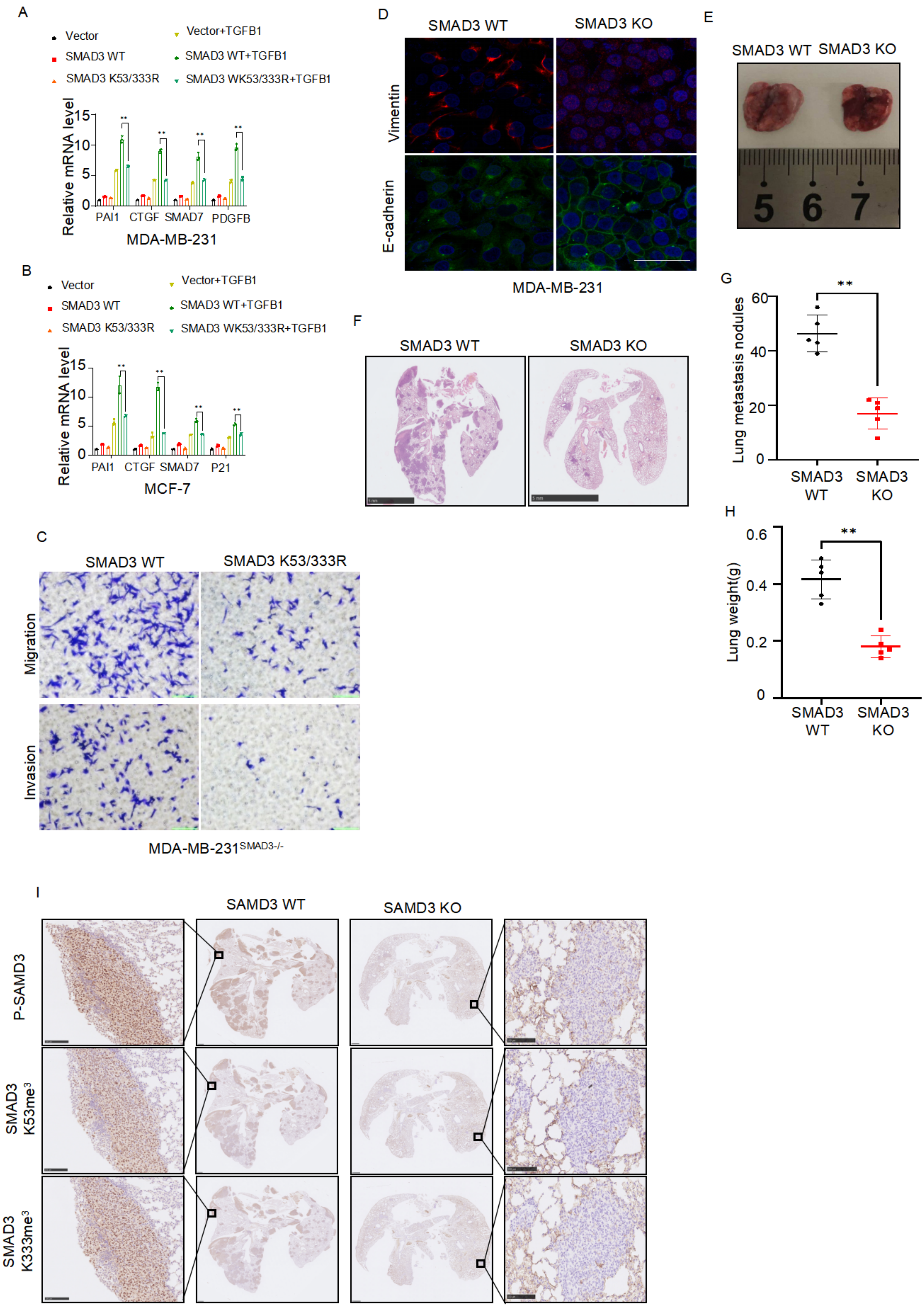
A
Vector
SMAD3 WT
SMAD3 K53/333R
Vector+TGFB1
SMAD3 WT+TGFB1
SMAD3 WK53/333R+TGFB1
Relative mRNA level
PAI1
CTGF
SMAD7
PDGFB
MDA-MB-231
B
Vector
SMAD3 WT
SMAD3 K53/333R
Vector+TGFB1
SMAD3 WT+TGFB1
SMAD3 WK53/333R+TGFB1
Relative mRNA level
PAI1
CTGF
SMAD7
P21
MCF-7
C
SMAD3 WT
SMAD3 K53/333R
Migration
Invasion
MDA-MB-231SMAD3-/-
D
SMAD3 WT
SMAD3 KO
Vimentin
E-cadherin
MDA-MB-231
E
SMAD3 WT
SMAD3 KO
F
SMAD3 WT
SMAD3 KO
G
Lung metastasis nodules
SMAD3 WT
SMAD3 KO
H
Lung weight(g)
SMAD3 WT
SMAD3 KO
I
SAMD3 WT
SAMD3 KO
P-SAMD3
SMAD3 K53me3
SMAD3 K333me3

Figure.S3

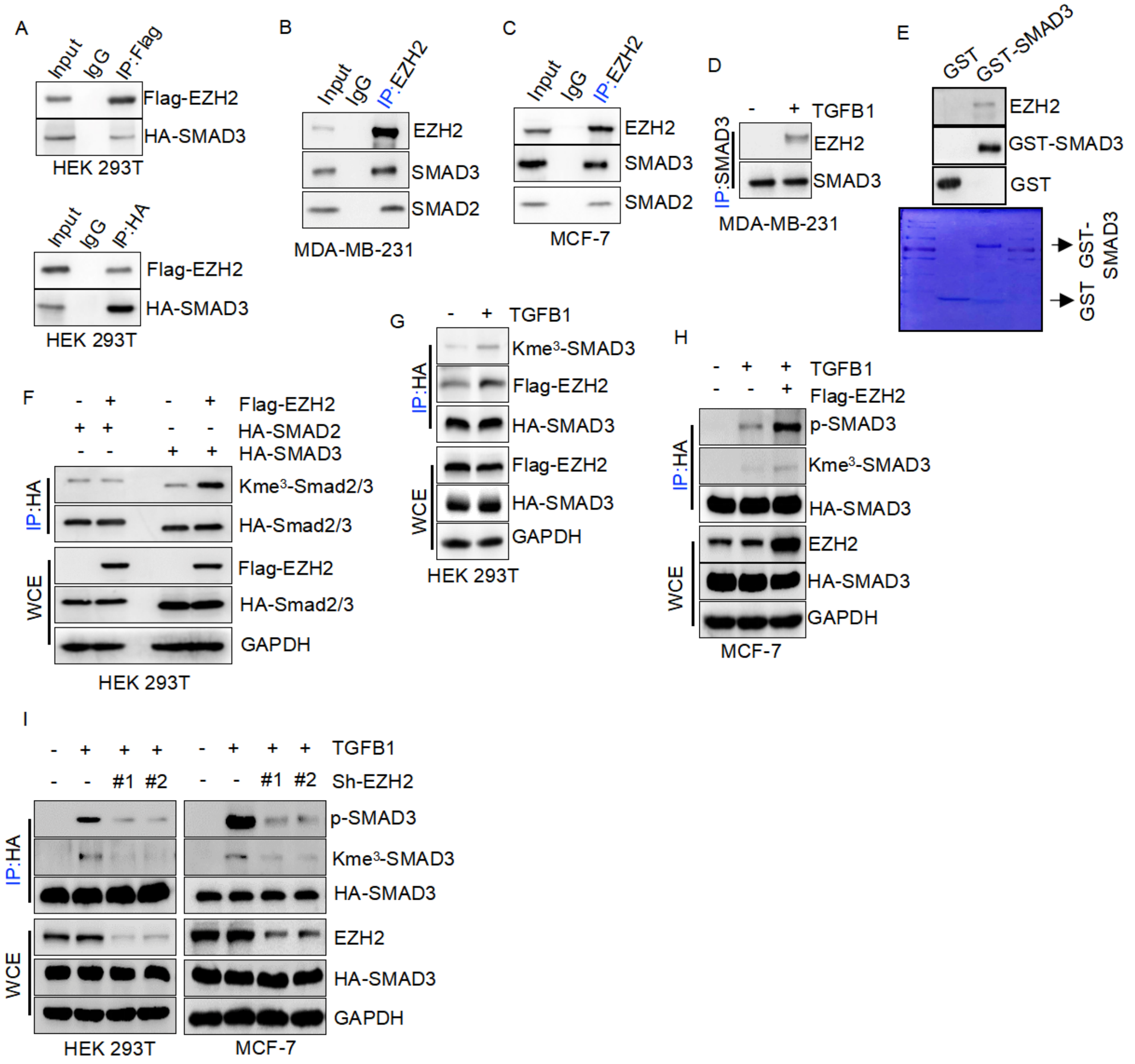
A
Input
IgG
IP:Flag
Flag-EZH2
HA-SMAD3
HEK 293T
Input
IgG
IP:HA
Flag-EZH2
HA-SMAD3
HEK 293T
B
Input
IgG
IP:EZH2
EZH2
SMAD3
SMAD2
MDA-MB-231
C
Input
IgG
IP:EZH2
EZH2
SMAD3
SMAD2
MCF-7
D
- + TGFB1
IP:SMAD3
EZH2
SMAD3
MDA-MB-231
E
GST
GST-SMAD3
EZH2
GST-SMAD3
GST
GST-SMAD3
GST
F
- + - + Flag-EZH2
+ + - - HA-SMAD2
- - + + HA-SMAD3
IP:HA
Kme3-Smad2/3
HA-Smad2/3
WCE
Flag-EZH2
HA-Smad2/3
GAPDH
HEK 293T
G
- + TGFB1
IP:HA
Kme3-SMAD3
Flag-EZH2
HA-SMAD3
WCE
Flag-EZH2
HA-SMAD3
GAPDH
HEK 293T
H
- + + TGFB1
- - + Flag-EZH2
IP:HA
p-SMAD3
Kme3-SMAD3
HA-SMAD3
WCE
EZH2
HA-SMAD3
GAPDH
MCF-7
I
- + + + - + + + TGFB1
- - #1 #2 - - #1 #2 Sh-EZH2
IP:HA
p-SMAD3
Kme3-SMAD3
HA-SMAD3
WCE
EZH2
HA-SMAD3
GAPDH
HEK 293T
MCF-7

Figure.S4

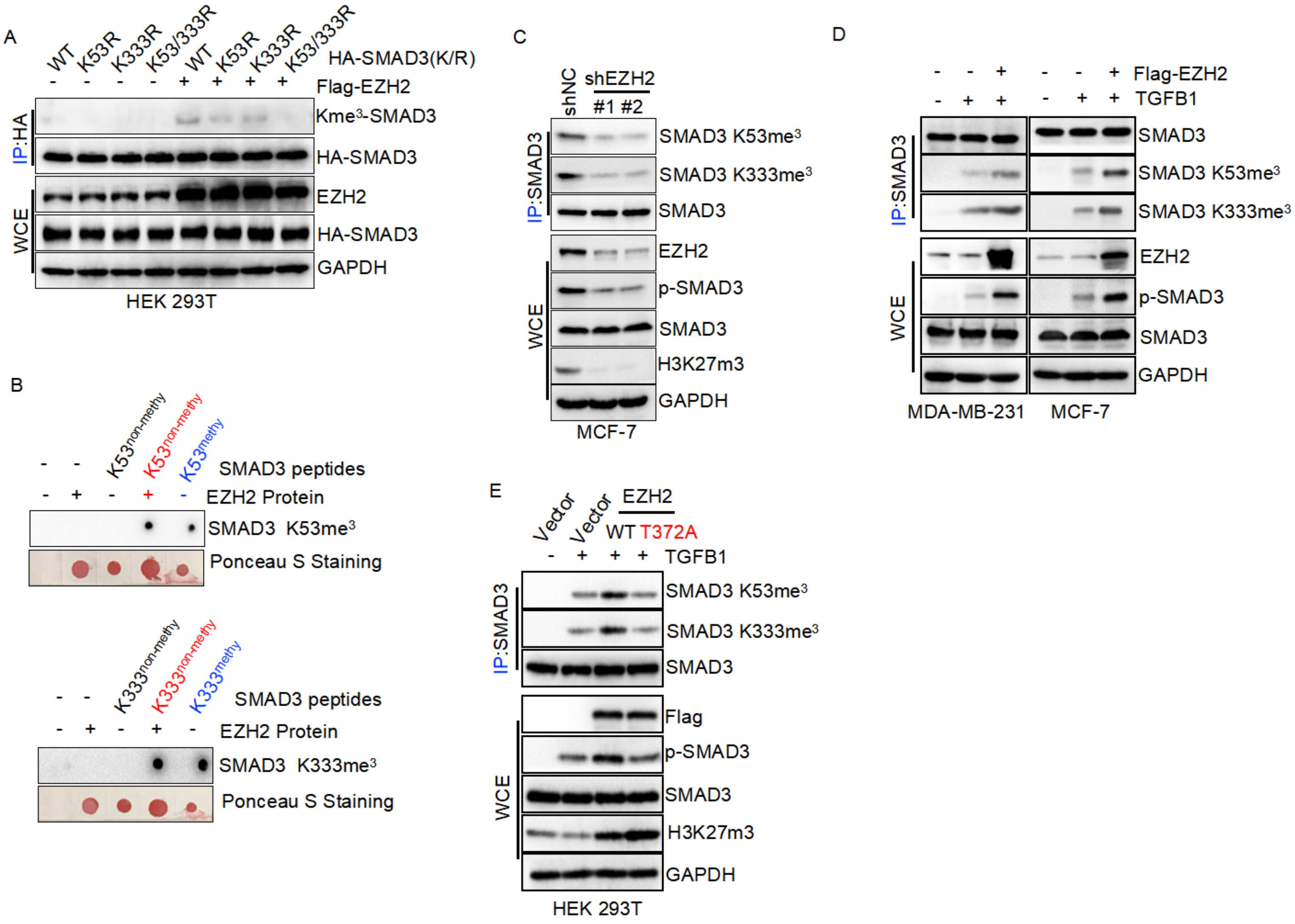
A
WT K53R K333R K53/333R WT K53R K333R K53/333R HA-SMAD3(K/R)
- - - - + + + + Flag-EZH2
IP:HA
Kme3-SMAD3
HA-SMAD3
WCE
EZH2
HA-SMAD3
GAPDH
HEK 293T
B
K53non-methy K53non-methy K53methy SMAD3 peptides
- + - + - EZH2 Protein
SMAD3 K53me3
Ponceau S Staining
K333non-methy K333non-methy K333methy SMAD3 peptides
- + - + - EZH2 Protein
SMAD3 K333me3
Ponceau S Staining
C
shNC shEZH2 #1 #2
IP:SMAD3
SMAD3 K53me3
SMAD3 K333me3
SMAD3
WCE
EZH2
p-SMAD3
SMAD3
H3K27m3
GAPDH
MCF-7
D
- - + - - + Flag-EZH2
- + + - + + TGFB1
IP:SMAD3
SMAD3
SMAD3 K53me3
SMAD3 K333me3
WCE
EZH2
p-SMAD3
SMAD3
GAPDH
MDA-MB-231 MCF-7
E
Vector Vector EZH2 WT T372A
- + + + TGFB1
IP:SMAD3
SMAD3 K53me3
SMAD3 K333me3
SMAD3
WCE
Flag
p-SMAD3
SMAD3
H3K27m3
GAPDH
HEK 293T

Figure.S5

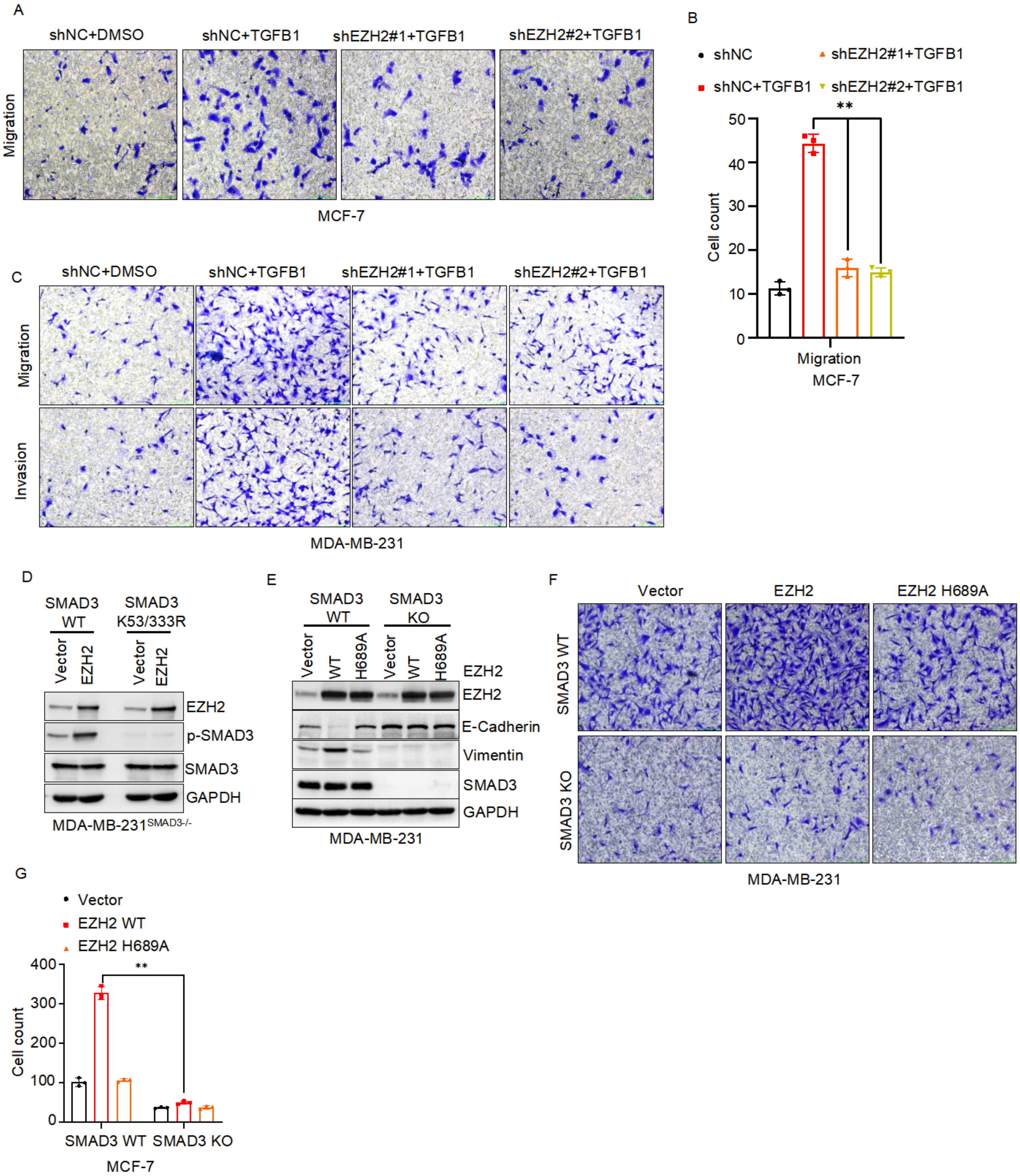
A
shNC+DMSO
shNC+TGFB1
shEZH2#1+TGFB1
shEZH2#2+TGFB1
Migration
MCF-7
B
shNC
shNC+TGFB1
shEZH2#1+TGFB1
shEZH2#2+TGFB1
**
50
40
30
20
10
0
Cell count
Migration
MCF-7
C
shNC+DMSO
shNC+TGFB1
shEZH2#1+TGFB1
shEZH2#2+TGFB1
Migration
Invasion
MDA-MB-231
D
SMAD3 WT
SMAD3 K53/333R
Vector
EZH2
Vector
EZH2
EZH2
p-SMAD3
SMAD3
GAPDH
MDA-MB-231 SMAD3-/-
E
SMAD3 WT
SMAD3 KO
Vector
WT
H689A
Vector
WT
H689A
EZH2
EZH2
E-Cadherin
Vimentin
SMAD3
GAPDH
MDA-MB-231
F
Vector
EZH2
EZH2 H689A
SMAD3 WT
SMAD3 KO
MDA-MB-231
G
Vector
EZH2 WT
EZH2 H689A
**
400
300
200
100
0
Cell count
SMAD3 WT
SMAD3 KO
MCF-7

Figure.S6

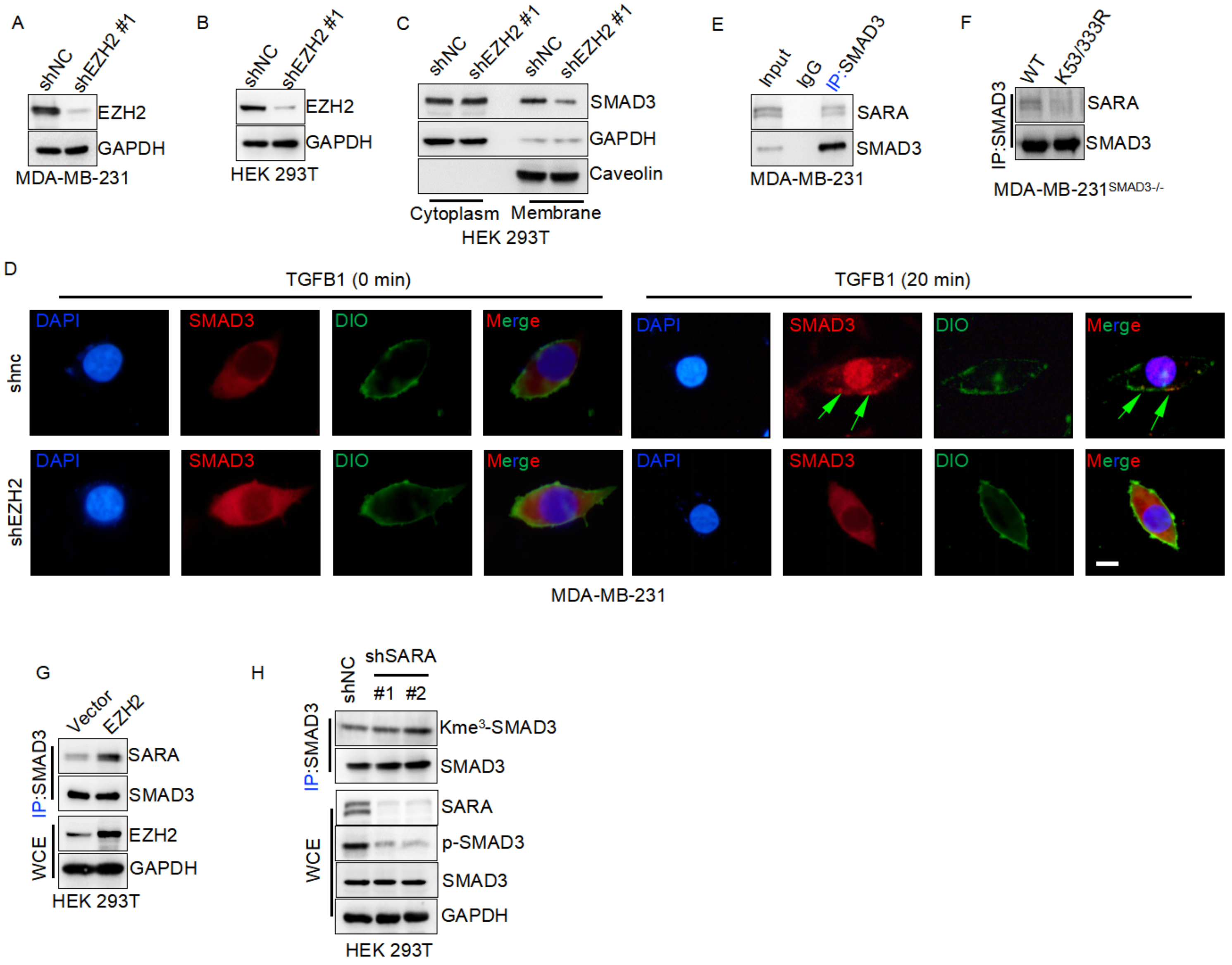
A
shNC
shEZH2 #1
EZH2
GAPDH
MDA-MB-231
B
shNC
shEZH2 #1
EZH2
GAPDH
HEK 293T
C
shNC
shEZH2 #1
shNC
shEZH2 #1
SMAD3
GAPDH
Caveolin
Cytoplasm
Membrane
HEK 293T
E
Input
IgG
IP:SMAD3
SARA
SMAD3
MDA-MB-231
F
IP:SMAD3
WT
K53/333R
SARA
SMAD3
MDA-MB-231SMAD3-/-
D
TGFB1 (0 min)
TGFB1 (20 min)
shnc
shEZH2
DAPI
SMAD3
DIO
Merge
MDA-MB-231
G
Vector
EZH2
IP:SMAD3
WCE
SARA
SMAD3
EZH2
GAPDH
HEK 293T
H
shNC
shSARA
#1
#2
IP:SMAD3
WCE
Kme3-SMAD3
SMAD3
SARA
p-SMAD3
SMAD3
GAPDH
HEK 293T

Figure.S7

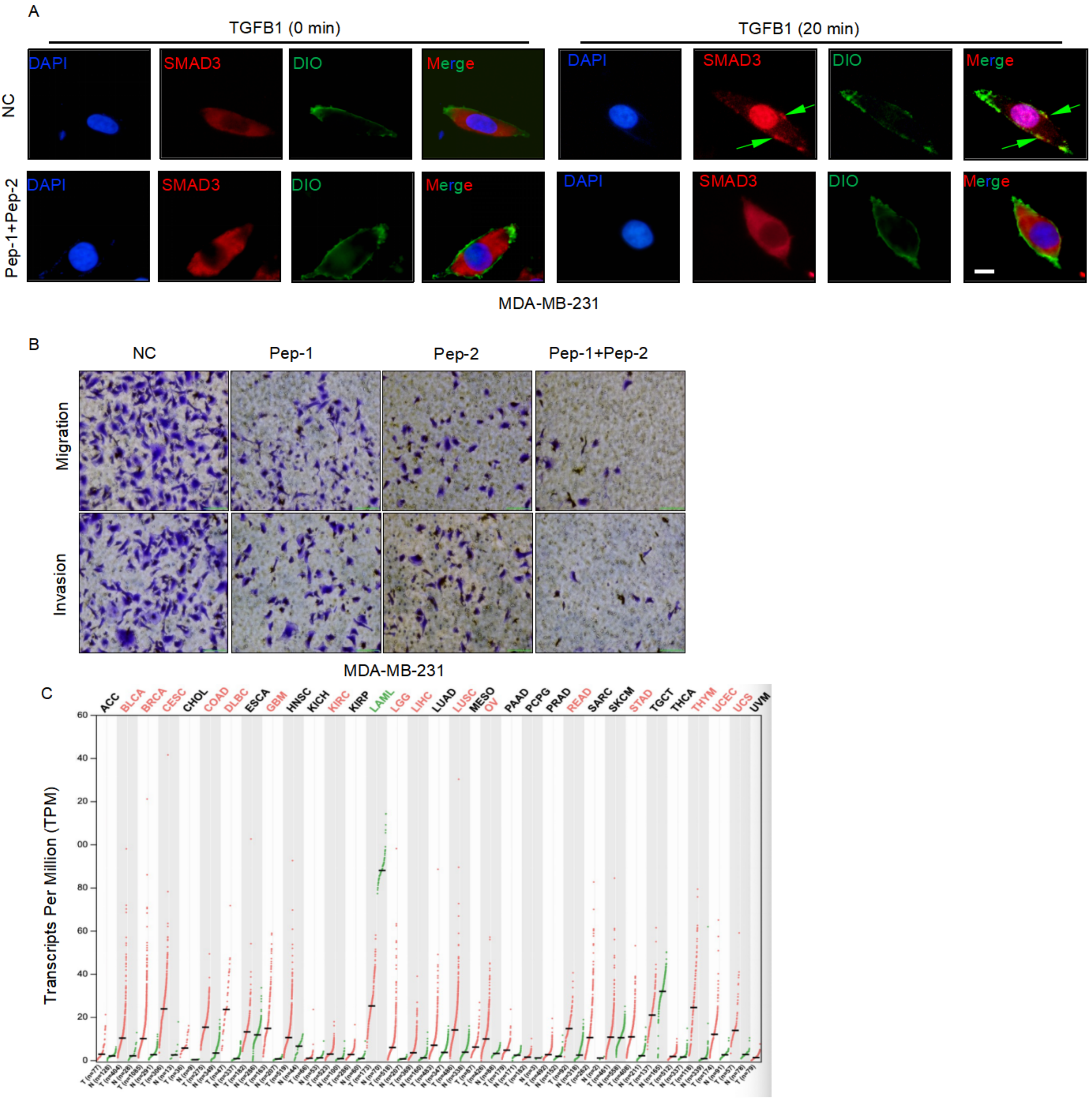

ACC,Adrenocortical carcinoma; BLCA, Bladder Urothelial Carcinoma; BRCA, Breast invasive carcinoma; CESC, Cervical squamous cell carcinoma and endocervical adenocarcinoma; CHOL, Cholangio carcinoma; COAD, Colon adenocarcinoma; DLBC, Lymphoid Neoplasm Diffuse Large B-cell Lymphoma; ESCA, Esophageal carcinoma; GBM, Glioblastoma multiforme; HNSC, Head and Neck squamous cell carcinoma; KICH, Kidney Chromophobe; KIRC, Kidney renal clear cell carcinoma; KIRP, Kidney renal papillary cell carcinoma; LAML, Acute Myeloid Leukemia; LGG, Brain Lower Grade Glioma; LIHC, Liver hepatocellular carcinoma; LUAD, Lung adenocarcinoma; LUSC, Lung squamous cell carcinoma; MESO, Mesothelioma; OV, Ovarian serous cystadenocarcinoma; PAAD, Pancreatic adenocarcinoma; PCPG, Pheochromocytoma and Paraganglioma; PRAD, Prostate adenocarcinoma; READ, Rectum adenocarcinoma; SARC, Sarcoma; SKCM, Skin Cutaneous Melanoma; STAD, Stomach adenocarcinoma; TGCT, Testicular Germ Cell Tumors; THCA, Thyroid carcinoma; THYM, Thymoma; UCEC, Uterine Corpus Endometrial Carcinoma; UCS, Uterine Carcinosarcoma; UVM, Uveal Melanoma

Figure.S8

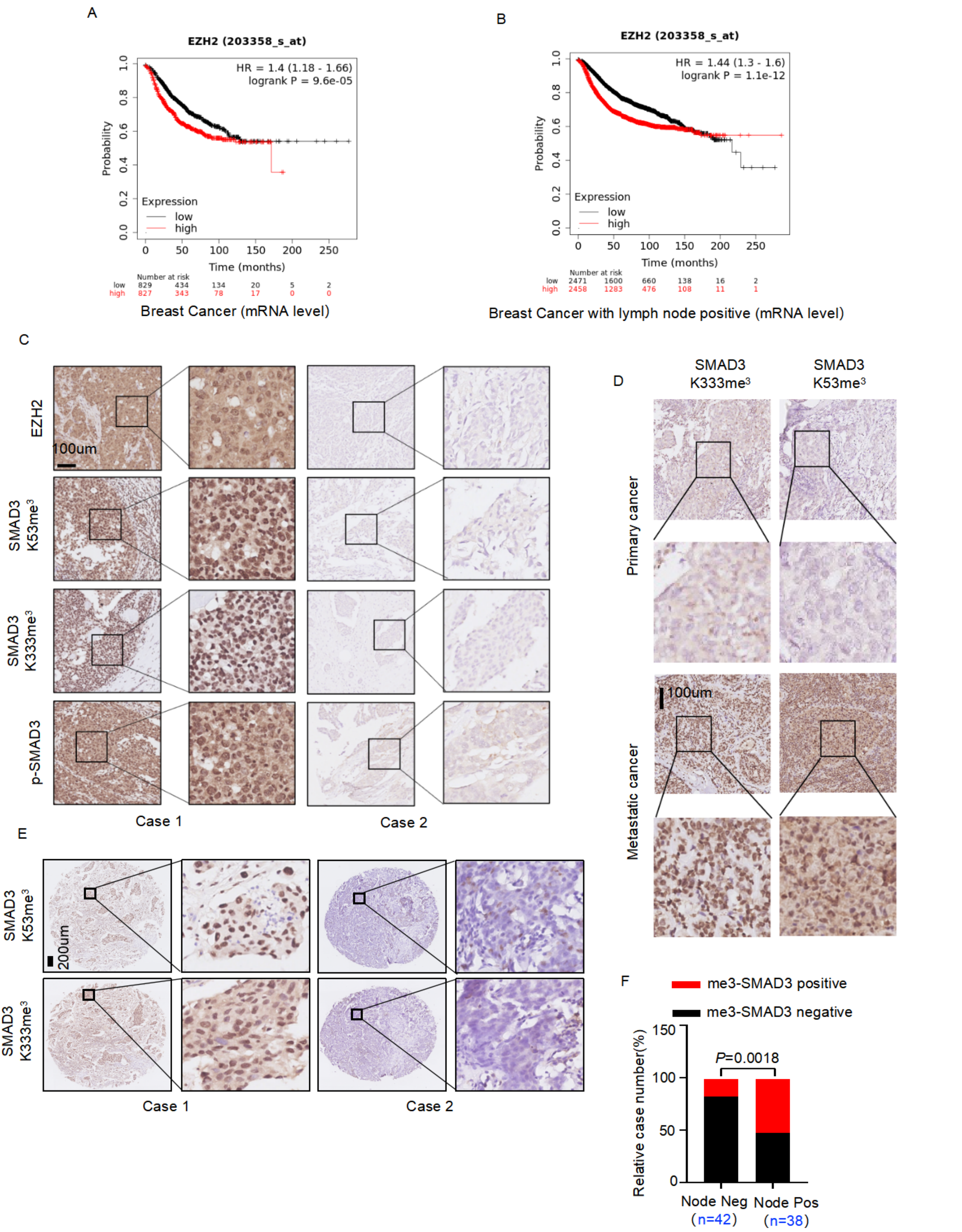
A
EZH2 (203358_s_at)
HR = 1.4 (1.18 - 1.66)
logrank P = 9.6e-05
Probability
0.0 0.2 0.4 0.6 0.8 1.0
Expression
low
high
0 50 100 150 200 250
Time (months)
Number at risk
low 829 434 134 20 5 2
high 827 343 78 17 0 0
Breast Cancer (mRNA level)
B
EZH2 (203358_s_at)
HR = 1.44 (1.3 - 1.6)
logrank P = 1.1e-12
Probability
0.0 0.2 0.4 0.6 0.8 1.0
Expression
low
high
0 50 100 150 200 250
Time (months)
Number at risk
low 2471 1600 660 138 16 2
high 2458 1283 476 108 11 1
Breast Cancer with lymph node positive (mRNA level)
C
EZH2
100um
SMAD3 K53me3
SMAD3 K333me3
p-SMAD3
Case 1
Case 2
D
SMAD3 K333me3
SMAD3 K53me3
Primary cancer
Metastatic cancer
100um
E
SMAD3 K53me3
200um
SMAD3 K333me3
Case 1
Case 2
F
me3-SMAD3 positive
me3-SMAD3 negative
Relative case number(%)
0 50 100 150
P=0.0018
Node Neg (n=42)
Node Pos (n=38)
